# Genetic and codon usage bias analyses of polymerase genes of equine influenza virus and its relation to evolution

**DOI:** 10.1186/s12864-017-4063-1

**Published:** 2017-08-23

**Authors:** Bidhan Ch. Bera, Nitin Virmani, Naveen Kumar, Taruna Anand, S Pavulraj, Adam Rash, Debra Elton, Nicola Rash, Sandeep Bhatia, Richa Sood, Raj Kumar Singh, Bhupendra Nath Tripathi

**Affiliations:** 1 National Research Centre on Equines, Sirsa Road, Hisar, Haryana India; 2 National Institute of High Security Animal Diseases, Hathai Kheda Dam Road, Anand Nagar, Bhopal, Madhya Pradesh India; 3 Animal Health Trust, Lanwades Park, Kentford, Newmarket, Suffolk CB8 7UU UK; 4 Indian Veterinary Research Institute, Izatnagar, Bareilly, Uttar Pradesh India

**Keywords:** Equine influenza virus, H3N8, Polymerase genes, Codon usage bias, Evolution

## Abstract

**Background:**

Equine influenza is a major health problem of equines worldwide. The polymerase genes of influenza virus have key roles in virus replication, transcription, transmission between hosts and pathogenesis. Hence, the comprehensive genetic and codon usage bias of polymerase genes of equine influenza virus (EIV) were analyzed to elucidate the genetic and evolutionary relationships in a novel perspective.

**Results:**

The group - specific consensus amino acid substitutions were identified in all polymerase genes of EIVs that led to divergence of EIVs into various clades. The consistent amino acid changes were also detected in the Florida clade 2 EIVs circulating in Europe and Asia since 2007. To study the codon usage patterns, a total of 281,324 codons of polymerase genes of EIV H3N8 isolates from 1963 to 2015 were systemically analyzed. The polymerase genes of EIVs exhibit a weak codon usage bias. The ENc-GC3s and Neutrality plots indicated that natural selection is the major influencing factor of codon usage bias, and that the impact of mutation pressure is comparatively minor. The methods for estimating host imposed translation pressure suggested that the polymerase acidic (PA) gene seems to be under less translational pressure compared to polymerase basic 1 (PB1) and polymerase basic 2 (PB2) genes. The multivariate statistical analysis of polymerase genes divided EIVs into four evolutionary diverged clusters - Pre-divergent, Eurasian, Florida sub-lineage 1 and 2.

**Conclusions:**

Various lineage specific amino acid substitutions observed in all polymerase genes of EIVs and especially, clade 2 EIVs underwent major variations which led to the emergence of a phylogenetically distinct group of EIVs originating from Richmond/1/07. The codon usage bias was low in all the polymerase genes of EIVs that was influenced by the multiple factors such as the nucleotide compositions, mutation pressure, aromaticity and hydropathicity. However, natural selection was the major influencing factor in defining the codon usage patterns and evolution of polymerase genes of EIVs.

**Electronic supplementary material:**

The online version of this article (doi:10.1186/s12864-017-4063-1) contains supplementary material, which is available to authorized users.

## Background

Equine influenza outbreaks are of major concern throughout the world. The vaccines often fail to protect horses, especially in the case of newly emerged equine influenza A (H3N8) viruses owing to antigenic variation of haemagglutinin (HA) protein [1, 2]. Thus due to its importance for vaccine strain selection [3, 4] most evolutionary dynamic studies have been focused on HA gene [5–7]. Since their first isolation in 1963 (Miami/63), EIV H3N8 have diverged due to variation in the HA gene viz.*,* Pre-divergent, Eurasian and American (evolved into currently circulating Florida sub-lineage clade1 & 2) [5, 7, 8]. The evolution of internal genes of EIV H3N8 in Greek outbreak [9] and evidence of species-jumping of H3N8 from horse to canine [10–12]; and pigs [13] emphasizes the role of internal genes, especially polymerase genes in replication, transcription and host adaptation.

The three largest genome segments of EIVs encode polymerase heterotrimeric proteins namely basic protein 1 (PB1), basic protein 2 (PB2) and acidic protein (PA). These subunits together with the nucleoprotein form the complex which is accountable for viral RNA transcription and replication [14–16]. Genome segment 2 encodes the PB1 subunit, the central component of the polymerase complex with RNA polymerase activity; PB1 interacts directly with PA and PB2 subunits [17–21]. Segment 2 also encodes another protein, PB1-F2, from an alternate reading frame of PB1 mRNA [22, 23]. PB1-F2 is known to increase virulence through the induction of cell death, promoting inflammation and up-regulating viral polymerase activity [24–26]. Genome segment 1 encodes the PB2 subunit, a key player in the initiation of viral transcription through cap-snatching activity in conjunction with endonuclease function of PA subunit [17, 27–29]. Finally, segment 3 encodes the PA subunit, which is responsible for generalized proteolysis of viral and host proteins [30, 31] and plays a role in virus assembly [32]. Segment 3 also encodes a second protein called PA-X [33], which modulates viral pathogenesis [34, 35]. In addition to their basic functions in viral genome expression, the polymerase proteins play a pivotal role in host adaptation and viral pathogenesis [34, 36, 37]. Several mutations in the polymerase subunits of avian and human influenza viruses have been implicated in enhancing virus replication and adaptation to various hosts [38–42]. However, the factors dictating the genetic changes of polymerase genes of EIV H3N8 especially in terms of codon usage bias patterns have not been explored in detail.

The codon usage pattern has a significant role in the evolution of viruses. Several studies have testified the species-specific synonymous codon usage bias [43–45], which shows preference for certain codons encoding for the same amino acids [46]. Such preference for codon usage offers evolutionary force for determining the overall fitness of the virus influencing various cellular processes [47–49]. So far, limited studies have highlighted the factors responsible in shaping the codon usage bias in different influenza viruses. For instance, while mutation pressure plays a key role in shaping the codon usage bias in H1N1 (human influenza virus) [50] and H9N2 (avian influenza virus) [51], natural selection primarily dictates the bias of synonymous codon usage for EIV [52]. One study on the PB1-F2 gene of EIV showed that mutational bias along with selection pressure and gene length influenced the codon usage pattern [53]. Furthermore, the codon usage bias analysis provides a different perspective regarding virus evolution studies in comparison to phylogenetic studies. Nevertheless, comprehensive analysis of codon usage pattern of polymerase of EIVs has not been elucidated so far.

Thus the present study focuses on comprehensive analysis of genetic evolution, synonymous codon usage pattern and factors involved in shaping the codon usage pattern of three polymerase genes across the lineages of EIV H3N8 strains circulating worldwide from 1963 to 2015. Combining the codon usage bias and traditional phylogenetic analyses of polymerase genes of EIV H3N8 will help in understanding the novel perspective of molecular evolution dynamics of EIVs.

## Results

### Amino acid variations in the phylogenetic clusters of polymerase genes

The deduced amino acid sequences of polymerase genes of EIV H3N8 were aligned and compared against A/equine/Richmond/07 isolate - representative strain of Florida clade 2 sub-lineage of EIV H3N8. The detailed amino acid substitutions observed in PA, PA-X, PB1, PB1-F2 and PB2, proteins encoded by the polymerase genes of EIV H3N8 are presented in additional file (see Additional file 1: Table S1a, S1b, S1c, S1d & S1e).

In the PA protein, four amino acid substitutions (I62V, I270M, I432V & V/F450I) were found in EIVs from 1993 (A/equine/Newmarket/1/93) onwards which persisted in Florida clade 1 & 2 isolates (Table 1 and Additional file 1: Table S1a). Moreover, from 2002 onwards five amino acid substitutions (R213K, A337T, A343E, L345I & K353R) could be observed in all Florida clade 1 and 2 isolates except A/equine/Wisconsin/03, A/equine/Cheshire/06 and A/equine/Linconshire/06. Florida clade 2 isolates from A/equine/Richmond/07 onwards showed six amino acid changes (E64D, M86I, E237K, S/N321 N, A476T & K626R) which are not seen in A/equine/Newmarket/5/03 isolate. The PA sequences of all Asian isolates till 2013 showed almost 100% similarities with A/equine/Richmond/07 isolate from Europe except one amino acid change (K to R/G) at position 158.

**Table 1:** Amino acid substitutions in the polymerase genes of EIV H3N8 among different lineages/clades

Genes	Residue no.	Pre-divergent	Eurasian	Florida clade 1	Florida clade 2
PA	62	I	V	V	V
	64	E	E	E	D
	86	M	M	M	I
	158	K	K	K	R/G/K
	213	R	R	K	K
	231	A	V	V	V
	237	E	E	E	K
	269	R	K	K	K
	270	I	I/M	M	M
	321	S/N	S	S	N
	337	A	A	T	T
	343	A	A	E	E
	345	L	L	I	I
	353	K	K	R	R
	354	T/I	T/D	I	I
	388	S	N	S	S
	409	S	S	N/S	S
	432	I	I	V	V
	450	V	V/F	I/V	I
	476	A	A	A	T
	532	L	F	F	F
	626	K	K	K	R
PA-X	213	G	G	S	S
PB1	114	V	V	I	I
	119	V	V	V	M
	154	D	D	G	G
	198	K	R	R	R
	221	A	A	T/A	T
	317	M	M	I	I
	329	Q	Q	Q	R
	377	D	D	D	E
	618	E	E	E	D
	738	E	E/D	D	D
PB1-F2	4	G	G	G	E
	18	T	T	I	I
	21	R	R	K	R
	41	R	R	H	H
	79	R	R	R	Q
PB2	12	S	S	L	L
	105	T	T	T	A
	251	R	R/K	R/K	K
	344	V	M	M	M
	511	V	I	I	I
	590	G	S	S	S

In PA-X protein, amino acid substitutions at positions 62, 64, 86, & 158 were similar to what was observed in complete PA protein (Additional file 1: Table S1b). However, one specific consensus point mutation (G213S) was observed in Florida clade 1 & 2 isolates from 2002 onwards except A/equine/Cheshire/06 & A/equine/Linconshire/06 isolates. However, a non-sense point mutation was observed at position 210 in A/equine/Richmond/07 isolate. These results in a truncated PA-X protein, which was reported previously [54].

In PB1 protein, lineage - specific amino acid changes were found (Table 1 and Additional file 1: Table S1c). Four point mutations (V119 M, Q329R, D377E & E618D) were observed subsequently in all Florida clade 2 isolates from Europe (A/equine/Richmond/07) and Asia (A/equine/Xinjiang/07). Furthermore, four consensus amino acid changes (V114I, D154G, A221T & M317I) were noticed in EIVs of Florida sublineage clades (1&2) from 1993/2002 onwards.

In the PB1-F2 protein, two point mutations (T18I in A/equine/Newmarket/1/93 & R41H in A/equine/New York/99) appeared during 1993–99 and were subsequently observed in all Florida clade 1 & 2 isolates (Additional file 1: Table S1d). However, a Florida clade 1 specific mutation (R21K) was continuously observed in EIVs from 2003 onwards. Furthermore, two amino acid substitutions (G4E & R79Q), first observed during the 2007 outbreaks in Europe (A/equine/Richmond/07) and Asia (A/equine/Almaty/07 & A/equine/Xinjiang/07) were subsequently found in all Florida clade 2 isolates except A/equine/Perthshire/09.

In PB2 protein, two consensus amino acid changes (T105A & R251K) were observed in Florida clade 2 lineage (Table 1) wherein, R251K substitution started in Athens/03 isolate (data not shown) and other substitution (T105A) found in isolates subsequently from 2007 outbreak in Europe (A/equine/Richmond/07) and Asia (A/equine/Xinjiang/07). Three amino acid substitutions (V344 m, V511I & G590S) were found consistently in EIVs from 1985 onwards. All Indian isolates had two unique amino acid substitutions (V606I & R380K) (Additional file 1: Table S1e).

### Phylogenetic analysis

**Fig. 1:**
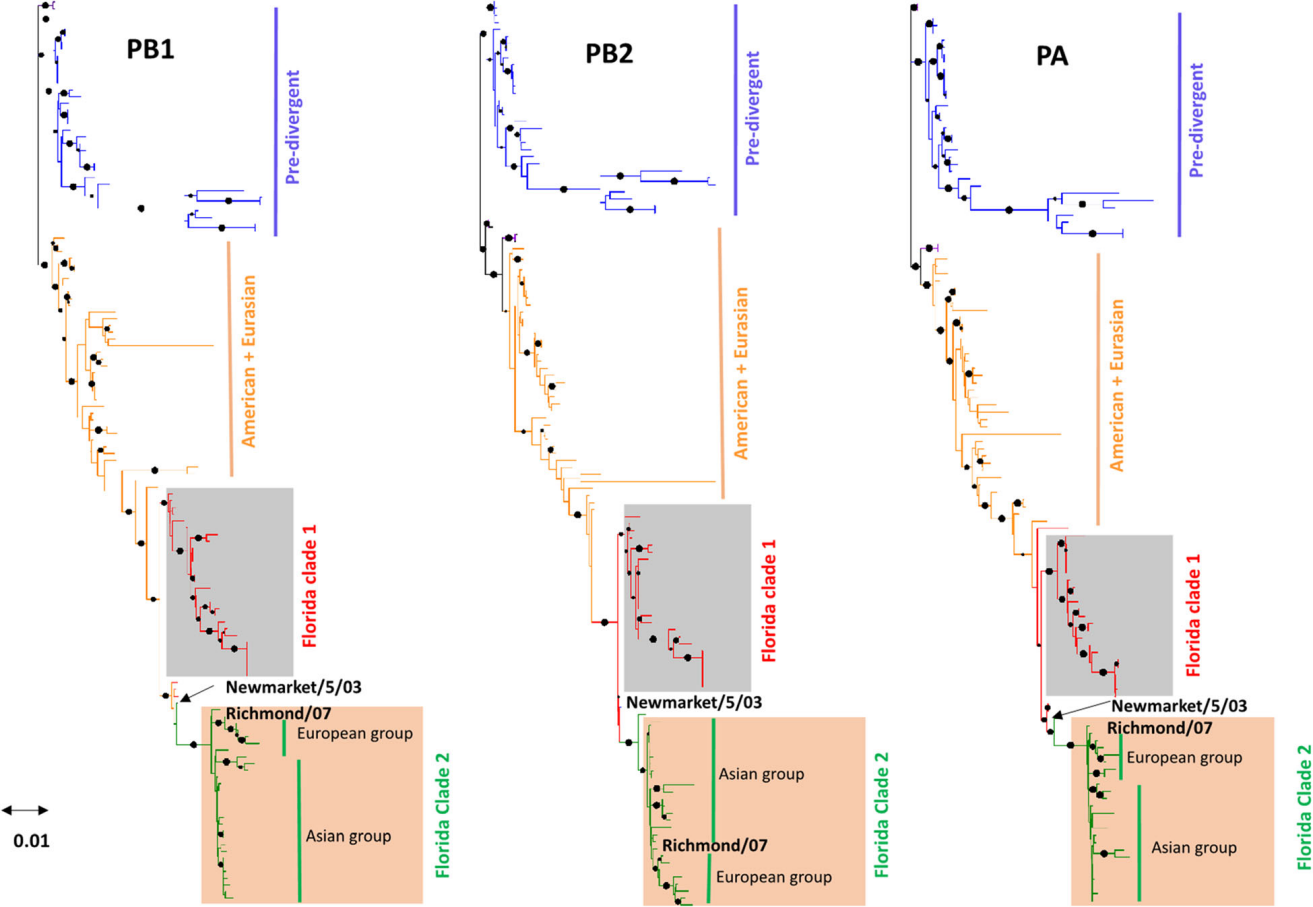
Phylogenetic analyses of polymerase genes of EIVs. The trees were generated by the maximum likelihood model implemented in the software MEGA 5.0. The reliability of the trees was assessed by bootstrap with 1000 replications. The bootstrap values higher than 0.7 are highlighted with solid black dots. The distinct Florida clade 1 and clade 2 sublineages of EIVs have been represented by colored boxes

The phylograms of polymerase genes broadly showed that the segment 3 (PA) is more evolutionarily divergent as compared to segment 1 (PB2) & 2 (PB1). The phylogram of PA gene divided EIVs circulating globally into four major groups: Florida clade (1& 2), American, Eurasian and Pre-divergent viruses (Fig. 1) corresponding to those observed in the HA1gene phylogram [5]. The topogram of PA tree also revealed time scale specific grouping of EIVs, wherein A/equine/Newmarket/1/93 was the closest sister lineage of Florida clade-1 & 2 lineages and A/equine/Newmarket/5/03 was the parent isolate of the Florida clade 2 sub-lineage (Fig. 1). Furthermore, in Florida clade 2 lineage, from A/equine/Richmond/07 onwards, EIVs formed a separate group along with 2007 isolates from China (A/equine/Xinjiang/07) and Kazakhstan (A/equine/Almaty/07). The PA-X phylogenetic tree depicted a similar pattern of EIV H3N8 grouping into four major groups as observed for PA gene (Additional file 2). However, two exceptions were found, where A/equine/Shropshire/10 isolate grouped close to A/equine/Newmarket/5/03 in Florida clade 2 lineage and A/equine/Snailwell/98 isolate clustered between A/equine/Newmarket/1/93 and other Florida clade 1 members.

The phylogram of PB1 gene divided EIV H3N8 into Florida clade 1 & 2, America + Eurasian and Pre-divergent lineages (Fig. 1). The EIVs from A/equine/Richmond/07 onwards were divided into two separate groups – the isolates from Asian countries including China, Kazakhstan & India formed one group and European isolates formed other group. The phylogeny of PB1 also revealed that A/equine/Newmarket/1/93 isolate although clustered separately with A/equine/Cheshire/06, but broadly grouped under Eurasian lineage (Fig. 1). The evolutionary pattern of PB1-F2 gene was different from the PB1 gene, where EIVs grouped into Florida clade 2, Florida clade 1+ clade 2, American, Eurasian and pre-divergent (Additional file 2). The A/equine/Richmond/07 isolate clustered closely with recent European isolates and A/equine/Newmarket/5/03 isolate grouped with Florida clade 1 viruses.

The phylogram of PB2 gene classified EIVs into separate groups - Florida clade 1 & 2, American + Eurasian and Pre-divergent, however A/equine/Newmarket/5/03 clustered in between Florida clade 1 and American lineages (Fig. 1). In Florida clade 2 lineage, A/equine/Richmond/07 isolate clustered separately from all other isolates. Similar to PB1 gene, EIVs in Florida clade 2 lineage again subdivided into two separate sub-groups from A/equine/Richmond/07 onwards. The Asian isolates from China, Kazakhstan & India formed one sub-group and European isolates formed other sub-group within Florida clade 2.

### Nucleotide composition affects the codon usage bias

The influence of compositional constraints on the codon usage was assessed by computing the nucleotide compositions of the polymerase genes of EIV and correlating these with the principal axes generated on correspondence analysis (COA) which is used to study the trends in codon usage variations. The mean compositions (%) of nucleotides A (PA = 34.27 ± 0.36, PB1 = 35.10 ± 0.42, PB2 = 34.58 ± 0.48) and G (PB1 = 22.47 ± 0.39, PB2 = 24.50 ± 0.44, U in PA = 24.0 ± 0.31) were highest, and C (%) being the lowest (PA = 18.63 ± 0.44, PB1 = 20.37 ± 0.24, PB2 = 18.94 ± 0.19). The nucleotides at the third positions of synonymous codons (A3, G3, U3, and C3) showed slight variations in the compositional trends. The mean values of A3 (PA = 32.24 ± 0.84, PB1 = 33.09 ± 1.33, PB2 = 36.44 ± 1.35) continued to be highest. Other nucleotides (G3, U3, and C3) in PA and PB2 showed similar trends as their individual nucleotides, while PB1 displayed almost analogous mean values for C3 (22.44 ± 0.67), G3 (22.30 ± 1.21), and U3 (22.17 ± 0.54).

Moreover, correlation of different nucleotide compositions with the two principal axes of COA was performed. In the case of PB1, axis 1 has distinct positive correlation with G3 (*r* = 0.959, *p* < 0.0001), GC3s (*r* = 0.954, *p* < 0.0001) and negative correlation with A3 (*r* = −0.956, *p* < 0.0001). Furthermore, the Enc (Effective number of codons) values were computed to assess the degree of codon usage bias in the polymerase coding sequences of EIV. There is significant positive correlation between ENc and GC3s (*r* = 0.613, *p* < 0.0001), while ENc has positive (*r* = 0.668, *p* < 0.0001) and negative (*r* = −0.678, *p* < 0.0001) correlation with axis 1 and 2, respectively. The case of PB2 is quite different, where axis 1 has distinct positive correlation with A3 (*r* = 0.852, *p* < 0.0001) and distinct negative correlation with G3 (*r* = −0.871, *p* < 0.0001), C3 (*r* = −0.821, *p* < 0.0001), GC3s (*r* = −0.880, *p* < 0.0001), and ENc (*r* = −0.840, *p* < 0.0001). Similarly, axis 2 also showed distinct correlations with these nucleotide compositions in an opposite polarity pattern (Additional file 3). In the case of PA, axis 1 has distinct positive correlation with A3 (*r* = 0.910, *p* < 0.0001), and distinct negative correlation with C3 (*r* = −0.892, *p* < 0.0001), GC3s (*r* = −0.840, *p* < 0.0001), ENc (*r* = −0.659, *p* < 0.0001). These results demonstrate that compositional constraints indeed affect codon usage bias in all the polymerase genes of EIV, but to a different extent.

### Relative synonymous Codon usage (RSCU) analysis

The RSCU analysis determines the patterns of synonymous codons usage without the confounding influence of amino acid compositions. We analyzed RSCU values of polymerase genes of EIVs and compared them with their clinical host species (Table 2). The RSCU values of the majority of preferred and non-preferred codons fell between 0.6 and 1.6. Amongst the 18 most frequently used codons in EIVs, fourteen in PA genes (eight A-ended; six U-ended), thirteen in PB1 (nine A-ended; four U-ended) and twelve codons in PB2 (eight A-ended; four U-ended) were A/U-ended, while remaining were G/C-ended codons. We furthermore analyzed the over- and under-represented codons and found that almost all the over-represented codons (RSCU ≥1.6) in PA [CCA (Pro), ACA (Thr), CGA (Arg), GGA (Gly)]; PB1 [CCA (Pro), ACA (Thr), GCA (Ala), CGA/CGG (Arg)] and PB2 [CCA (Pro), ACA (Thr), GCA (Ala), UCA (Ser), UGC (Cys), GGA (Gly)] were A-ended, while nearly all the under-represented codons (RSCU ≤0.6) were G-ended except CCU (Pro), CGU (Arg), UGU (Cys) in PA; CGU (Arg) in PB1 and UUA (Leu), UCU (Ser), UGU (Cys) in PB2 genes. Furthermore, the RSCU values of 59 sense codon from all the polymerase genes of EIV were compared with RSCU values of their host species- horse, donkey and dog. None of the over-represented codon in any of the polymerase genes was common to their host species. Nevertheless, five codons [CCG (Pro), ACG (Thr), GCG (Ala), UCG (Ser) and CGU (Arg)] were under-represented in both polymerase genes and their host species. There were some differences in the RSCU values of polymerase genes but the overall trend is somewhat similar (Additional file 4).

**Table 2:** The Relative synonymous codon usage (RSCU) patterns of polymerase genes of EIVs and their host species

Amino acid	Codon	PB2	PB1	PA	Horse	Donkey	Dog	Amino acid	Codon	PB2	PB1	PA	Horse	Donkey	Dog
Phe	UUU	0.98	0.63	1.22	0.83	0.88	0.82	Ser	UCA	**2.2**	1.58	1.18	0.80	0.83	0.81
	UUC	1.02	1.38	0.77	1.17	1.12	1.16		UCG	*0.43*	*0.17*	*0.15*	*0.34*	*0.24*	*0.38*
Leu	UUA	*0.55*	0.72	1.08	*0.33*	*0.24*	*0.35*		AGU	0.76	0.68	1.25	0.86	1.10	0.89
	UUG	1.45	1.29	0.92	0.72	0.82	0.68		AGC	1.24	1.32	0.74	1.48	1.27	1.56
	CUU	1.16	0.65	1.35	0.73	0.80	0.67	Arg	AGA	1.4	1.57	1.13	1.30	1.49	1.20
	CUC	0.88	0.79	1.05	1.32	**1.63**	1.25		AGG	0.6	*0.43*	0.87	1.32	**1.86**	1.32
	CUA	0.95	1.11	0.66	*0.34*	*0.18*	*0.37*		CGU	0.63	*0.00*	*0.57*	*0.55*	*0.49*	*0.46*
	CUG	1	1.44	0.92	**2.56**	**2.33**	**2.45**		CGC	0.99	*0.48*	1.13	1.15	0.79	1.26
Ile	AUU	1.31	0.91	1.24	0.92	*0.58*	0.96		CGA	0.98	**1.91**	**2.1**	0.61	0.74	0.67
	AUC	*0.52*	0.85	0.83	**1.66**	**1.95**	**1.61**		CGG	1.4	**1.61**	*0.17*	1.08	0.62	1.31
	AUA	1.18	1.24	0.93	*0.42*	*0.47*	*0.45*	Cys	UGU	*0.01*	1.19	*0.59*	0.89	0.62	0.85
Val	GUU	0.76	0.91	0.96	0.60	0.64	*0.58*		UGC	**1.99**	0.81	1.4	1.11	1.38	1.10
	GUC	0.66	1.12	0.63	1.08	1.42	1.1	His	CAU	1.4	1.11	0.92	0.81	0.89	0.78
	GUA	1.18	1.08	1.04	*0.35*	*0.29*	*0.42*		CAC	0.6	0.89	1.07	1.19	1.11	1.22
	GUG	1.4	0.89	1.34	**1.97**	**1.65**	**1.98**	Gln	CAA	1.25	1.24	1.25	*0.52*	0.84	*0.50*
Pro	CCU	1.1	1.13	*0.39*	1.19	0.83	1.08		CAG	0.75	0.76	0.74	1.48	1.16	1.46
	CCC	0.96	0.75	0.85	1.38	**1.60**	1.47	Asn	AAU	1.2	1.15	0.97	0.84	0.66	0.87
	CCA	**1.79**	**1.66**	**2.16**	0.97	1.06	1.05		AAC	0.8	0.85	1.02	1.16	1.34	1.12
	CCG	*0.14*	*0.47*	*0.58*	*0.45*	*0.51*	*0.51*	Lys	AAA	1.37	1.32	1.33	0.79	0.79	0.79
Thr	ACU	0.83	1.07	0.77	0.94	0.82	0.89		AAG	0.63	0.89	0.67	1.21	1.21	1.13
	ACC	0.93	0.85	1.11	1.58	**1.77**	1.58	Asp	GAU	1.15	1.27	1.25	0.83	0.84	0.86
	ACA	**1.85**	**1.89**	**2**	0.96	0.79	1.05		GAC	0.85	0.73	0.74	1.17	1.16	1.09
	ACG	*0.39*	*0.19*	*0.1*	*0.52*	0.61	*0.53*	Glu	GAA	1.36	1.34	1.16	0.76	0.84	0.79
Ala	GCU	0.94	1.03	1.26	1.05	1.20	1		GAG	0.64	0.66	0.83	1.24	1.16	1.23
	GCC	0.93	0.94	0.81	**1.72**	**1.74**	**1.78**	Gly	GGU	0.7	0.74	0.84	0.65	0.87	0.65
	GCA	**1.79**	**1.75**	1.44	0.77	0.77	0.81		GGC	0.67	*0.5*	*0.49*	1.43	1.42	1.45
	GCG	*0.35*	*0.29*	*0.48*	*0.45*	*0.30*	*0.47*		GGA	**1.99**	1.53	**1.7**	0.95	0.85	1.02
Tyr	UAU	0.76	0.96	1.23	0.75	0.63	0.79		GGG	0.64	1.23	0.96	0.97	0.86	1.05
	UAC	1.24	1.04	0.76	1.25	1.37	1.15								
Ser	UCU	*0.53*	0.68	1.38	1.09	1.10	1.09								
	UCC	0.84	1.57	1.27	1.43	1.46	1.52								

Over- (RSCU ≥1.6) and under-represented (RSCU ≤0.6) codons are displayed in bold and italics, respectively

### Codon usage bias among polymerase genes varies and is not clade specific

The ENc values were calculated to estimate the magnitude of codon bias in the polymerase genes of EIV H3N8. The mean ENc values of PB1 (53.04 ± 0.63) was highest, followed by PA (52.12 ± 0.55) and PB2 (49.48 ± 0.88). The ENc values of PB2 were significantly lower compared to PB1 and PA (*p* < 0.0001). Furthermore, the ENc values were analyzed clade wise in each polymerase i.e. in four well defined clusters of EIV H3N8 (Pre-divergent, Eurasian, Florida clade 1 and Florida clade 2). There was no significant differences among these four clusters in a specific polymerase gene (*p* > 0.05). However, difference between the ENc values of PB1 and PB2 was more pronounced in clade 1 cluster (*p* < 0.001) compared to other clusters (*p* < 0.01). Similarly, we observed comparatively higher difference between the ENc values of PA and PB2 in clade 1&2 clusters (*p* < 0.01) compared to other clusters (*p* < 0.05).

### Mutation bias plays a minor role in the codon usage bias of polymerase genes of EIV H3N8

An ENc-plot is widely used to find whether mutation bias/mutation pressure influence the codon usage bias or not, whereas the Parity rule 2 (PR2) plot estimates the effects of mutation pressure and natural selection on the codon usage. Hence, to examine whether codon usage patterns in the polymerase genes of EIV have been governed by mutation pressure or not, ENc–GC_3s_ and PR2 plots were constructed. Firstly, a PR2 plot was constructed to examine whether the biased codon choices are restricted in highly biased protein-coding genes. For this, the relationship between purines (A and G) and pyrimidines (C and T) in the four fold degenerate codon families (Ala, Arg, Gly, Leu, Pro, Ser, Thr and Val) were determined. This plot showed that AU bias predominated in the fourfold degenerate codon families in all the polymerase genes coding sequences of EIV (Fig. 3). Furthermore elucidation was derived from the ENc–GC_3s_ plot. In this plot, all the polymerase genes of EIV H3N8 strains clustered below the expected ENc curve (Fig. 3). None of the strains of polymerase genes fell on the expected curve, which would have indicated a major role of mutation pressure in the coding sequences of polymerase genes. These under-curve clustering indicated that factors other than mutation pressure played a major role in the codon usage bias of polymerase genes of EIV H3N8.

### Natural selection predominates in shaping the codon usage patterns of polymerase genes of EIV H3N8

ENc–GC_3s_ plot analysis demonstrated the minor contribution of mutational pressures in shaping the codon usage patterns of the polymerase genes. We next sought to determine the magnitude of natural selection or mutation pressure in generating codon usage bias by constructing the neutrality plots, which determine the mutation-selection equilibrium in shaping the codon usage bias [55]. The distribution range of GC3 was narrow in all the polymerase genes, i.e. PA (40.2 to 47.8%), PB1 (42.5 to 50.3%), and PB2 (40 to 46.1%). There was noticeable correlation between GC1 and GC3 in PA (*r* = 0.280, *p* < 0.05) and PB1 (*r* = 0.610, *p* < 0.0001), which seemed to indicate the qualitative role of mutation pressure in codon usage bias.

In the neutrality plot analysis, a significant positive correlation was observed between the GC12 and GC3 values of PA (*r* = 0.61, *p* < 0.0001) and PB2 (*r* = 0.29, *p* = 0.001) genes of the EIV. However, the slopes of the regression line in PA and PB2 were calculated to be 0.0946 and 0.0335, respectively (Fig. 3). This indicates that the influence of direct mutation pressure for codon usage bias in PA and PB2 genes is only 9.46% and 3.35%, respectively. The contribution of natural selection in influencing the codon usage bias was high i.e. 90.54% in PA and 96.65% in PB2 genes. In the PB1 gene of EIV, a negative correlation (−0.332, *p* < 0.05) was observed, with a slope of −0.0133, and the mutation pressure and natural selection were determined to be 1.33% and 98.67%, respectively, again demonstrating the dominant influence of natural selection. Despite the observed correlation in all the polymerase genes, natural selection emerged as the dominant factor influencing the codon usage bias. Similar results were also obtained when selection pressure was executed on the polymerase genes, with PB1 (dN/dS = 0.082) being under the strongest purifying selection followed by PB2 (dN/dS = 0.129) and PA (dN/dS = 0.147).

### Translational selection affects codon usage bias

The influence of translational selection on codon usage bias is usually assessed based on how frequently preferred codons are recognized by the most abundant isoacceptor tRNAs of the host species. The most preferred codons (for each amino acid) were compared with their respective tRNA isotypes in equine cells. This analysis revealed that out of a total of nine, seven two-fold synonymous codon families (Asn, Lys, Asp, Glu, His, Gln, Cys) in PB1 & PB2 and six in PA (Phe, Lys, Asp, Glu, Gln, Tyr) were found to have ‘non-optimal codon–anticodon usage’ (Table 3). The remaining two (Phe and Tyr) had ‘optimal codon–anticodon usage’ in PB1 and PB2. But, these two aromatic amino acid (Phe and Tyr) in PA have their corresponding less frequent tRNA isotypes. We noticed optimal codon–anticodon usage, especially in hydrophobic amino acid (Val, Leu, Ile) of PA which is lacking in PB1 and PB2. Overall findings suggest that PA segment appears to have more adaptability to the tRNA pool of equine cells compared to PB1 and PB2 segments.

**Table 3:** Frequency of tRNA genes in equine cells for most preferentially used codons in polymerase genes of EIVs

AA	Preferentially used codons			tRNA isotypes in equine cells	Total count
	PB1	PB2	PA		
Ala	GCA	GCA	GCA	AGC (27), GGC (0), CGC (8), **TGC (10)**	45
Gly	GGA	GGA	GGA	ACC (0), GCC (10), CCC (8), **TCC (5)**	23
Pro	CCA	CCA	CCA	AGG (10), GGG (0), CGG (3), **TGG (7)**	20
Thr	ACA	ACA	ACA	AGT (9), GGT (0), CGT (3), **TGT (7)**	19
Val	GTC	GTC	GTG	AAC (12), **GAC (3)**, **CAC (16),** TAC (6)	37
Ser	TCA	TCA	TCT	**AGA (12)**, GGA (0), CGA (4), **TGA (4),** ACT (0), GCT (12)	32
Arg	AGA/CGG	CGA/CGG	CGA	ACG (10), GCG (1), **CCG (4)**, **TCG (5),** CCT (7), **TCT (6)**	33
Leu	TTG	CTG	CTT	**AAG (8),** GAG (0), **CAG (3)**, TAG (5), **CAA (6),** TAA (4)	26
Phe	TTC	TTC	TTT	**AAA (0)**, **GAA (13)**	13
Asn	AAT	AAT	AAC	**ATT (1), GTT (21)**	22
Lys	AAA	AAA	AAA	CTT (18), **TTT (15)**	33
Asp	GAT	GAT	GAT	**ATC (1),** GTC (10)	11
Glu	GAA	GAA	GAA	CTC (50), **TTC (11)**	61
His	CAT	CAT	CAC	**ATG (1), GTG (12)**	13
Gln	CAA	CAA	CAA	CTG (10), **TTG (6)**	16
Ile	ATT	ATA	ATT	**AAT (20),** GAT (0), **TAT (4)**	24
Tyr	TAC	TAC	TAT	**ATA (1), GTA (14)**	15
Cys	TGC	TGT	TGC	**ACA (0), GCA (26)**	26

Codons which are likely to be paired with respective anticodon are displayed in bold

### Trends in codon usage variations assessed by correspondence analysis

To examine the variations in the synonymous codons usage among the coding sequences of polymerase genes of EIVs, a multivariate statistical method, correspondence analysis (COA) was executed on the RSCU values of complete coding sequences and of codons. The first (ƒ´_1_) and second (ƒ´_2_) principal axes accounted for majority of data inertia (PB1: ƒ´_1_ = 41.4%, ƒ´_2_ = 15.3%; PB2: ƒ´_1_ = 40.1%, ƒ´_2_ = 25.5%; PA: ƒ´_1_ = 47.7%, ƒ´_2_ = 19.6%). COA analysis built on RSCU of codons revealed that codons in PB1 and PB2 were frequently distributed along the first (ƒ´_1_) and second (ƒ´_2_) principal axes, respectively (Fig. 4), while diffusely distributed in the case of PA. The PB1 and PA gene segments of EIV strains grouped into four defined clusters on both of the principal axes. Moreover, investigation in this type of clustering in PB1 and PB2 revealed that the strains clustered steadily in the format of clusters generated in the phylogenetic analysis, i.e. Pre-divergent, Eurasian, Florida clade 1 and clade 2 clusters (Fig. 4). This was not in the case of PA, where strains did not form these defined clusters and rather frequently distributed along the second (ƒ´_2_) principal axis.

### PB2 gene shows highest codon usage deoptimization for *Equus caballus*

The relative codon deoptimization index (RCDI) compares the similarity in codon usage of a given coding sequence with that of a reference genome. The RCDI values of each polymerase genes of EIV were computed to compare the similarity of the codon usage of these genes and the codon usage of *Equus caballus*. Mean RCDI values were highest for PB2 (1.43 ± 0.017) followed by PB1 (1.33 ± 0.013) and PA (1.32 ± 0.010) in relation to their clinical host, *Equus caballus*. Furthermore, the RCDI values were estimated cluster-wise in these polymerase genes to examine whether these variations are due to inherent properties of genes or arose during the course of evolution. We found that Florida clade 1 (PB1 = 1.37 ± 0.015, PB2 = 1.49 ± 0.020) and clade 2 (PB1 = 1.37 ± 0.003, PB2 = 1.47 ± 0.013) isolates displayed higher RCDI values than pre-divergent (PB1 = 1.28 ± 0.015, PB2 = 1.36 ± 0.014) and Eurasian (PB1 = 1.31 ± 0.019, PB2 = 1.38 ± 0.022) isolates in PB1 and PB2 genes (Fig. 5).

### *Equus caballus-*induced selection pressure is variable among EIV polymerase genes

A similarity (SiD) analysis was performed to examine whether codon usage patterns of *Equus caballus* influence the evolution of the codon usage patterns of the polymerase genes coding sequences. We found that *Equus caballus* exerted more selective pressure on the codon usage patterns of PB1 followed by PB2 and PA (Additional file 5).

### Amino acid hydropathicity plays a significant role in codon usage bias rather than aromaticity or genome length

The relationship among the hydropathicity, aromaticity, genome length, ENc and first two principal axes of COA were assessed through Spearman’s rank correlation analysis. Both General average hydropathicity (GRAVY) and aromaticity (AROMO) are indices of amino acid usage, and the variation in amino acid compositions can also influence the results of codon usage analysis. The GRAVY values of PB1 and PB2 had strong negative correlation with ENc (PB1: *r* = −0.287, *p* < 0.05; PB2: *r* = −0.388, *p* < 0.0001) and GC3s (PB1: *r* = −0.466, *p* < 0.0001, PB2: *r* = −0.393, *p* < 0.0001) (Additional file 6: Table S6a & S6b). The AROMO values had no significant correlation with ENc or GC3s or principal axes. Furthermore, the GRAVY values of PB1 and PB2 had negative strong correlation with axis 1 (*r* = −0.429, *p* < 0.0001) and axis 2 (*r* = −0.400, *p* < 0.0001), respectively suggesting the comparatively higher role of hydropathicity in influencing the codon usage bias in PB1. Likewise, in the PA gene segment, both AROMO and GRAVY values had strong significant correlation with ENc (GRAVY: *r* = 0.492, *p* < 0.0001, AROMO: *r* = −0.407, *p* < 0.0001), GC3s (GRAVY: *r* = 0.641, *p* < 0.0001, AROMO: *r* = −0.753, *p* < 0.0001) and principal axis 1 (GRAVY: *r* = −0.697, *p* < 0.0001, AROMO: *r* = 0.689, *p* < 0.0001) (Additional file 6: Table S6c) suggesting that both hydropathicity and aromaticity significantly influences the codon usage bias in PA gene segment.

## Discussion

The polymerase genes of EIVs like other gene segments undergo evolutionary changes. We showed that group - specific consensus amino acid substitutions occurred in polymerase genes among EIVs (H3N8) circulating since 1963, which led to divergence of EIVs. Furthermore, we analyzed codon usage pattern of these genes to understand the factors involved in evolution of EIV and their fitness towards host.

Currently EIVs belonging to clade 1 and 2 of Florida sublineage are circulating globally. Analysis of various isolates representing two lineages showed consistent 14 consensus amino acid changes from 2007 onwards. However, unavailability of sequences of polymerase genes between 2003 and 2007 obscures the exact origin of these changes. Availability of partial sequences of a Greek isolate (A/equine/Athens/03) with some of these mutations indicates their origin some time before [9]. Among polymerase proteins, PB2 is more conserved than the other two subunits (PA & PB1) of the RNP complex and interestingly, few stable point mutations in PB2 were found in all Asian isolates and A/equine/Richmond/07 isolate which are located in the cellular mRNA cap - binding site (318–483 amino acid residues) of this protein [28]. Similarly, the D377E change observed in PB1 of all Florida clade 2 EIVs may lead to alteration of stability of the protein as previously described [56]. An amino acid substitution G70E in PB1-F2 protein, which lies in the region essential for virus mediated apoptosis pathway [57–59], was found to be stable in all clade 2 viruses, but variable in European and Florida clade 1 lineages.

**Fig. 2:**
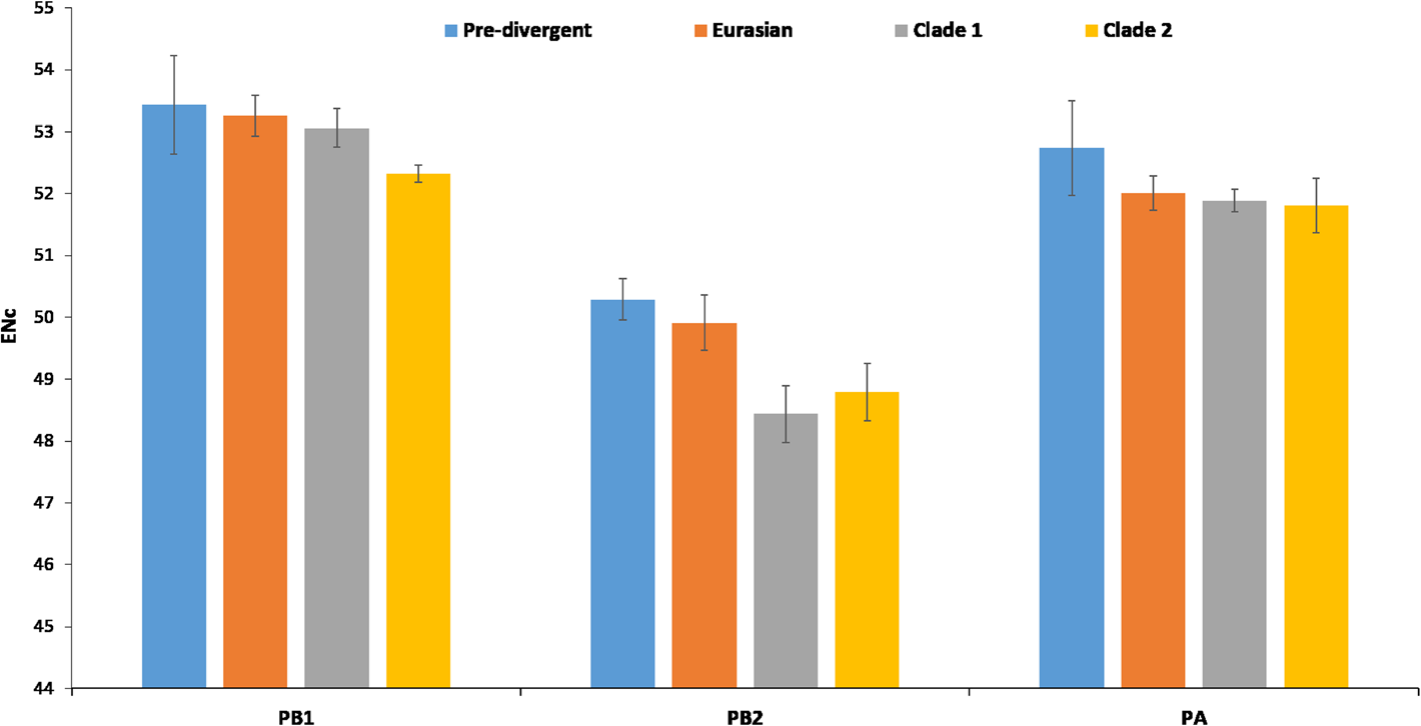
Comparison of phylogenetic derived clusters of EIVs polymerase genes based on their effective number of codon (ENc) values

**Fig. 3:**
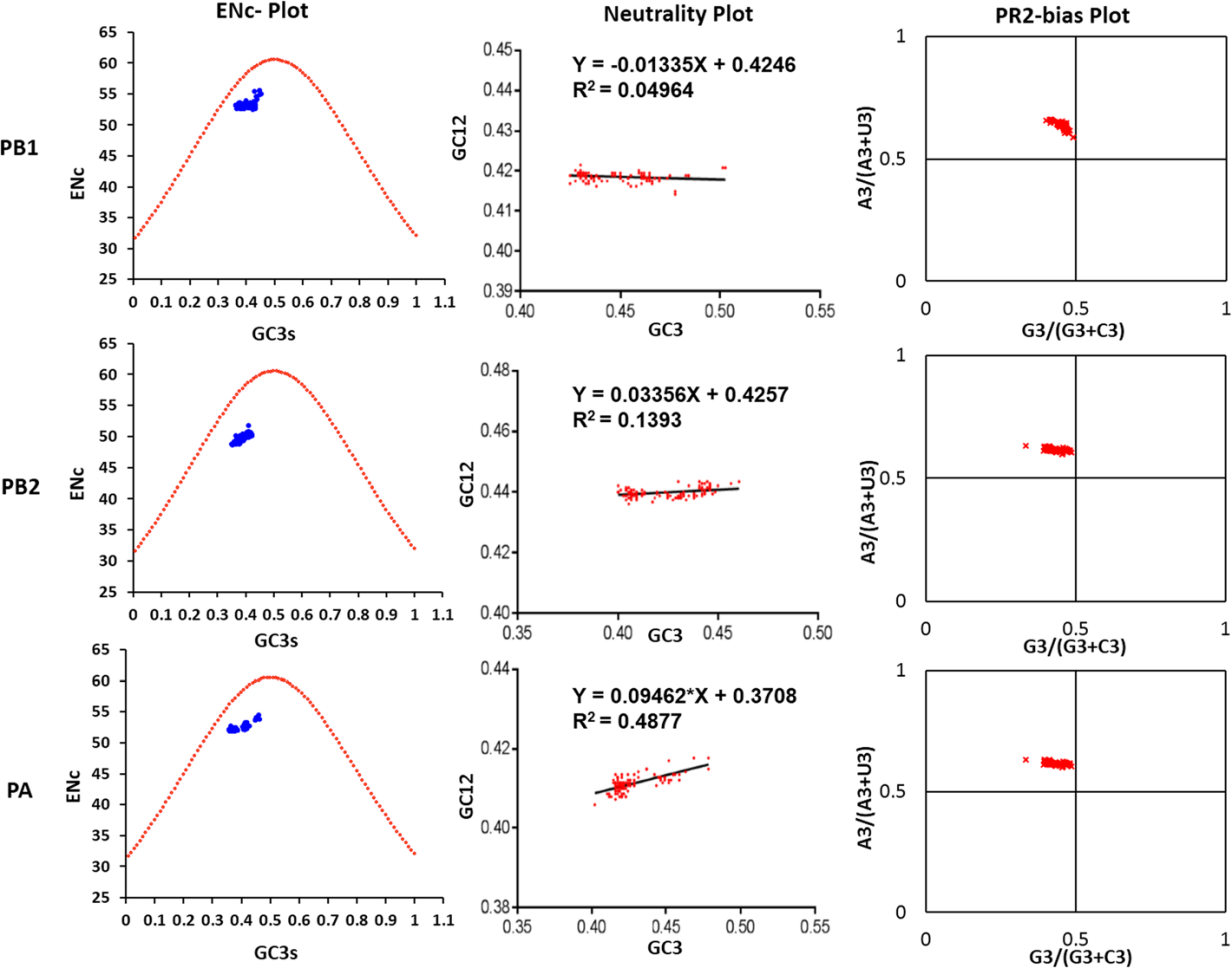
Magnitude of significant determining factors of codon usage bias in polymerase genes of EIVs. ENc-plot: the red dotted line represents the expected curve of positions of strains when the codon usage was only determined by the GC3s composition. Neutrality plot: the red dotted line is the linear regression of GC12 against GC3. PR2 bias plot: AU-bias [A3/(A3 + U3)] at the third codon position of the four-codon amino acids of entire genes were plotted against the GC-bias [G3/(G3 + C3)] and the centre of the plot represents no bias between the influence of the mutation pressure and natural selection

**Fig. 4:**
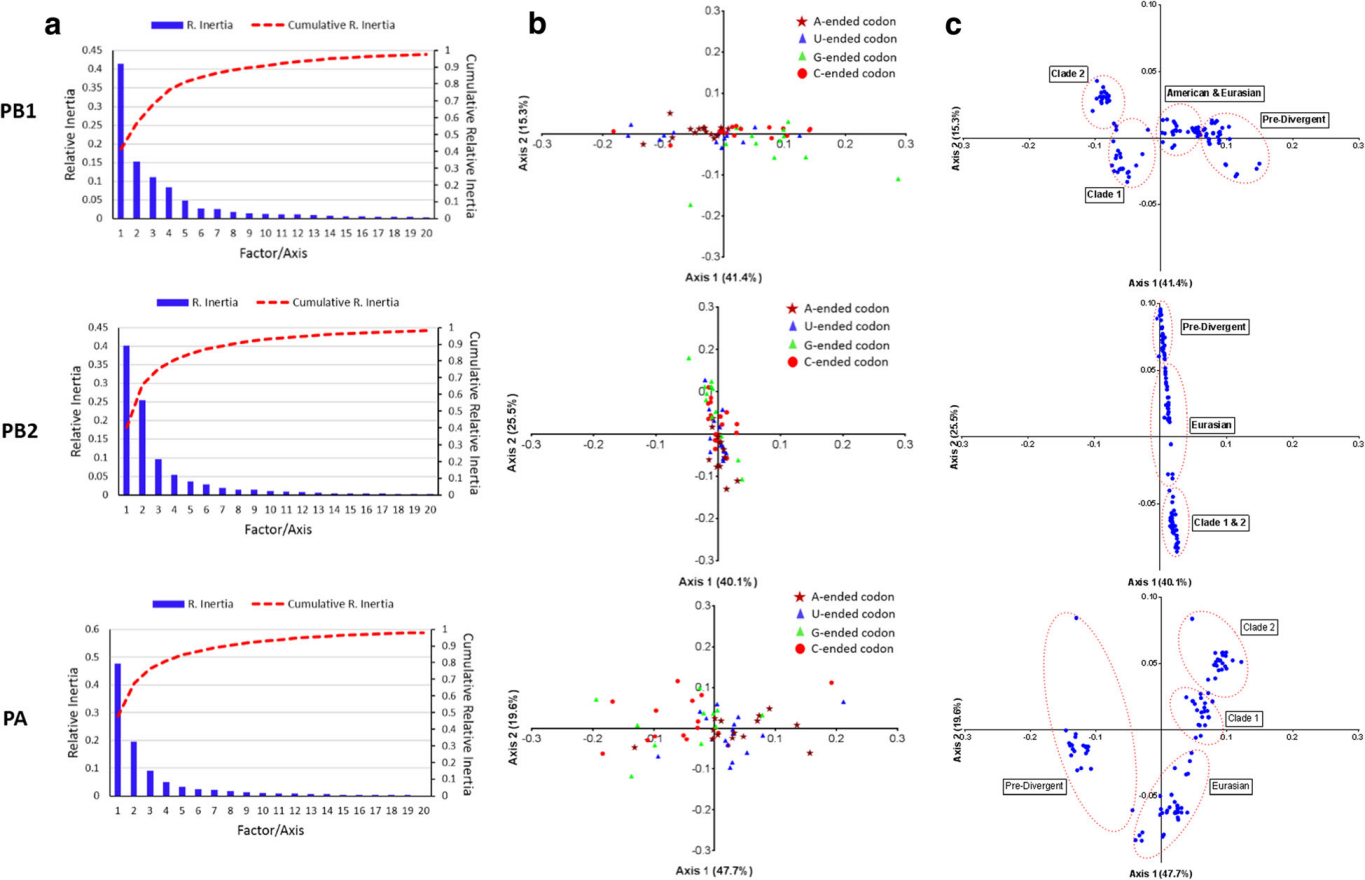
Correspondence analysis (COA) of the synonymous codon usage in EIVs polymerase genes. **a** COA generated axes contributions. The relative and cumulative inertia of the first 20 factors from a COA of the synonymous codon usage frequencies, (**b**) COA of the synonymous codon usage towards codons. This analysis was built on the RSCU values of the 59 synonymous codons. The positions of each codon were plotted on the first two-main-dimensional coordinates. Different base ended codons were color labelled. **c** COA of the synonymous codon usage in coding sequences of polymerase genes of EIV isolates. The positions of each polymerase genes of EIV isolates were plotted on the first two-main-axes

**Fig. 5:**
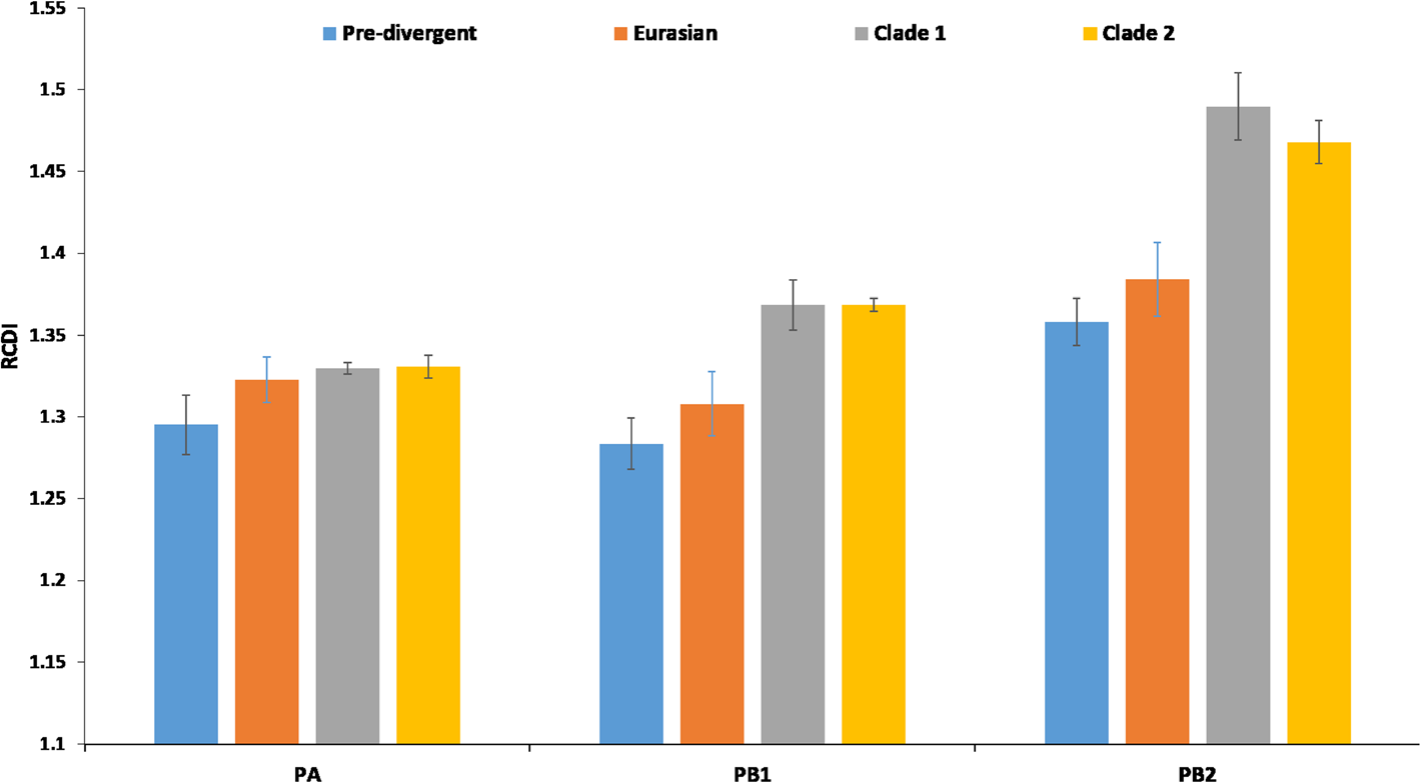
The RCDI analysis of phylogenetic derived clusters of EIVs polymerase genes in relation to their host species, *Equus caballus*

The surveillance of internal genes along with HA1 gene may help in better prediction of evolution pattern [60, 61] and severity of infections [62]. The phylogenetic analyses of the polymerase genes of EIVs circulating globally till 2015 have revealed differing evolutionary patterns (Figs. 1, 2, 3, 4 and 5). The isolates such as A/equine/Wisconsin/03 and A/equine/Newmarket/05/03 which represent clade 1 and clade 2 of Florida sublineage according to HA gene analysis did not group in the respective clades. The PA gene showed more evolutionary divergence and classified EIVs into specific lineages which, barring few isolates as mentioned above, followed the evolutionary pattern of HA1 and corroborate with the earlier analysis of equine influenza [63] and other influenza A viruses in various host animals [64, 65]. Our findings suggest that the divergence of Florida clade 2 sublineage of EIVs circulating in Asian region is occurring not only on the basis of stable point mutations in surface encoding HA protein [66], but also in the RNP complex proteins as well as Matrix (M1 and M2) protein [67]. The point mutations in polymerase complex subunits of influenza A viruses may have implication in host adaptation, virus replication and virulence as supported by several previous research findings [38, 68–70].

Various factors shaping the synonymous codon usage bias of the EIV polymerase genes were analyzed systematically. The correspondence analysis was carried out to assess the trends in codon usage variations of polymerase genes. This analysis has previously been used for demonstration of the evolutionary trends and classification of envelope glycoprotein genes or mitochondrial genome [71–74]. This revealed EIV strains to be grouped in well-defined clusters generated in the phylogenetic analysis in respect of PA and PB1 genes, whereas strains did not form defined clusters for PB2 gene.

The overall nucleotide composition affected the codon usage bias especially for codons ending with A/U, which is supported by the fact that over-represented codons (RSCU ≥1.6) were A-ended. Earlier studies on genome based codon usage bias analysis in different influenza viruses specifically H3N8 [52], H5N1 [75], H1N1 and H3N2 [50] also demonstrated a preference towards A- or U-ended codons. Interestingly, none of the over-represented codons in any of the polymerase genes was common to their host species. The ENc values indicated that overall codon usage bias was significantly lower in PB2 (49.48 ± 0.88) as compared to PB1 (53.04 ± 0.63) and PA (52.12 ± 0.55), while the difference was not significant amongst the four clusters of respective polymerase gene. ENc values dropped steadily during the course of evolution from 1963 to current isolates which may reduce competition between the virus and the host for utilizing cellular machinery and thus favour efficient replication of the virus [74, 76]. Similar low codon bias has also been observed in other RNA viruses such as Ebola virus [77], Zika virus [78], H1N1pdm IAV [79]; equine influenza virus H3N8 [52], Foot-and-Mouth Disease Virus [80]; H5N1 influenza virus [75] and equine infectious anaemia virus [81].

However, the low codon bias as revealed by the ENc does not reflect its underlying cause (mutation pressure and/or natural selection). To investigate this ENc–GC3s and PR-2 bias plots were constructed where the under-curve clustering of polymerase genes of EIVs showed that mutation pressure is not the sole factor that influenced the codon usage patterns of the EIV polymerase genes coding sequences. The magnitude of these factors in generating codon usage bias as determined by the neutrality plots, which indicated the predominant role of natural selection in influencing the codon usage bias in all the three polymerase genes (highest in PB1) of EIVs. However, previous studies reported that mutational pressure dominated in structuring the codon usage patterns of H1N1 pdm IAV [79], various RNA [82] and DNA viruses [83]. Contrastingly, PB1-F2, a protein encoded by an alternative open reading frame (ORF) of segment 2 of EIV having role in enhanced pathogenicity was governed by multiple factors such as mutation pressure, natural selection and gene length [53]. Host specific mutation bias was detected while comparing the human and avian influenza viruses [84].

The host imposed translational selection pressure also influenced the codon usage bias of the EIV polymerase genes which was assessed by comparing how most preferred codons are recognized by their respective tRNA isotypes in equine cells. Our previous studies showed that overall codon usage of EIVs does not seem to be well adapted to tRNA pool of equine cells [52]. However, more stringent analysis of polymerase genes in this study suggests that PA segment in comparison to PB1 and PB2 appears to have better adaptability to its clinical host, horse especially by utilizing the high copy number tRNA-anticodon for valine, serine leucine, aaparagine, histidine, isoleucine, cysteine in equine cells. Overall, the polymerase genes by virtue of this could capture the more copy of respective tRNA-anticodon in the equine cells and allow the virus to quickly replicate. However, overall adaptability of polymerase genes to tRNA pool of equine cells was moderate. Other determining parameters of natural selection driven codon bias, GRAVY and AROMO values (indices of amino acid usage) were estimated and correlated with two main axes of COA [85]. Interestingly, aromatic amino acid (Phe and Tyr) had ‘optimal codon–anticodon usage’ in PB1 and PB2, but not in PA. Also, both hydropathicity and aromaticity significantly influence the codon usage bias in PA gene segment, but only hydropathicity in both PB1 and PB2 gene segments.

The high RCDI in PB2 as compared to PB1 and PA genes indicates less adaptation to the codon usage towards highly expressed gene in *Equus caballus*. The increment in RCDI during the course of evolution in PB1 and PB2 furthermore suggests their less adaptability towards the host. This might lead to low replication rate for successful establishment of virus in its host with alternative codon usage patterns [86]. A similarity (SiD) index revealed that *Equus caballus* exerted more selective pressure on the codon usage patterns of PB1 as compared to PB2 and PA. Earlier studies also estimated low adaptability in HIV-1 [86] and H1N1pdm IAV in human cells [50]. Destite this, HIV-1 modulate the tRNA pool by selectively enhancement of tRNAs translating A-ending codons (86). This suggests that polymerase genes of EIV having moderate adaptability, may modulate tRNA pool and selectively enhance tRNAs translating A-ending codons for maintaining the efficient replication.

## Conclusions

The genetic analysis of the three polymerase genes of EIVs in the present study and matrix gene in our previous study revealed that clade 2 EIVs underwent major changes which led to emergence of a phylogenetically distinct group of EIVs originating from Richmond/1/07 (other sequences being unavailable). These consensus amino acid changes especially in clade 2 EIVs may have repercussions on pathologenicity and virulence of EIVs and needs further studies. The polymerase genes of EIVs exhibit a weak codon usage bias. Multiple factors such as nucleotide composition, mutation pressure, aromaticity and hydropathicity influenced the synonymous codon usage bias in polymerase genes, with natural selection being the major influencing factor.

## Methods

### Viruses and sequencing

The equine influenza virus (EIV) H3N8 isolates of Florida clade 2 sub-lineage viz. A/equine/Katra-Jammu/06/08, A/equine/Mysore/01/08 and A/equine/Ahmedabad/1/09 isolated by our laboratory from EI epizootic (2008–09) in India [66], were included in the study. The isolates were propagated in embryonated hen’s eggs. Viral RNA was extracted from 200 μl of allantoic fluid using a RNA extraction kit (QIAamp^(R)^ Viral RNA Mini Kit; Qiagen, Valencia, CA), cDNA synthesized using Uni12 primer [87] and polymerase genes were amplified using designed primers (Additional file 7). Amplifications of polymerase genes were carried out using 0.5 μl (3 U) of Hot-Start Taq DNA polymerase (Qiagen, Valencia, CA) in a 50 μl reaction volume with cycling conditions of 95 °C for 5 min followed by 34 cycles of 95 °C for 1 min, 52 °C for 1 min, 72 °C for 3 min and final extension at 72 °C for 10 min. All amplicons were cloned into pTZ57R/T vector (MBI Fermentas, Burlington, Canada) and three recombinant plasmids of each polymerase genes were sequenced commercially using Dye Deoxy Terminator Cycle Sequencing strategy from EuroFins Genomics India Pvt. Ltd. Bangalore, India.

### Sequence data

The full length nucleotide sequences of three polymerase genes of EIVs across different lineages reported worldwide between 1963 to 2015 were retrieved from the Influenza Virus Resource at the National Center for Biotechnological Information (http://www.ncbi.nlm.nih.gov/genomes/FLU/FLU.html). Few sequences from GISAID (www.gisaid.org) were also included in the present study in consultation with the Animal Health Trust, UK. The data set comprised 387 nucleotide sequences (124 of PA gene, 134 of PB1 gene & 129 of PB2 gene). The details of the EIV strains and accession numbers of sequences used in this study are provided in Additional file 8.

### Sequence and phylogenetic analyses

The consensus nucleotide sequences of 3 clones of each polymerase gene of Indian isolates were generated. Deposited in GenBank, NCBI and accession numbers are provided in Table S2. For comparative studies, the nucleotides as well as deduced amino acid sequences of these genes of EIV H3N8 circulating throughout the globe were aligned using ClustalW programme of MEGA5.0 software [88].

Phylogenetic trees of the polymerase genes were inferred using MEGA5.0 software employing the maximum likelihood model using nucleotide sequences including parameters of transitions and transversions of nucleotide substitutions, equal substitution rates among sites but heterogenous rate among lineages and gaps treated by pairwise-deletion [88]. The bootstrap analyses of the phylograms were carried out with 1000 replicates of dataset to determine the robustness of the individual nodes of the tree.

### Selection pressure analysis

Site specific selection pressure was estimated on three gene segments (PB1, PB2, and PA) of EIV using the HyPhy software implemented in the Datamonkey webserver [89]. The similar/duplicate sequences were excluded by this server. The best nucleotide substitution models, PA (012212), PB1 (012210), and PB2 (010210) were chosen based on Akaike Information Criterion (AIC) on a defined Neighbor-Joining (NJ) phylogenetic tree. We also performed GARD (Genetic Algorithm for Recombination Detection) to detect any recombination [90]. We compared four models, Single Likelihood Ancestor Counting (SLAC) model, Fixed Effect Likelihood (FEL) model, Mixed Effects Model of Evolution (MEME), and Fast Unbiased Bayesian Approximation (FUBAR) for estimation of sites under selection pressure [91–93]. The ratio of non-synonymous (dN) to synonymous (dS) substitutions per site (x = dN/dS) was estimated to know the strength of selection pressure. The sites with *p* values <0.1 for SLAC, FEL and MEME models, and a posterior probability >0.90 for FUBAR were accepted as candidates for selection.

### Nucleotide composition analysis

The nucleotide compositional parameters were calculated for polymerase genes of each EIV strain. These includes the frequencies of occurrence of each nucleotide (A %, U %, C %, and G %); each nucleotide at the third position of the synonymous codons (A3%, U3%, C3%, and G3%); G + C at the first (GC1), second (GC2), and third codon positions (GC3); G + C at the first and the second positions (GC12). The codons for Met (AUG), Trp (UGG) and termination codons (UAA, UGA, UAG) were removed from the analysis, as they were not expected to contribute in the codon usage bias.

### Relative synonymous codon usage (RSCU)

The RSCU values for all the coding sequences of polymerase genes of EIV were calculated to determine the patterns of synonymous codon usage without the confounding influence of amino acid composition. The RSCU value of a codon is measured as the ratio of its observed frequency to its expected frequency given that all codons for a particular amino acid are used equally [89]. RSCU values are not affected by sequence length and amino acid frequency since these factors are eliminated during the computation. The RSCU values were estimated as per equation given below [94]:

$$ RSCU\kern0.5em =\kern0.5em \frac{g_{ij}}{\underset{j}{\overset{ni}{\varSigma }}{g}_{ij}} ni $$

Where g_ij_ is the observed number of the i^th^ codon for the j^th^ amino acid which has n_i_ kinds of synonymous codons. Synonymous codons showing the RSCU values of <1.0 represent negative codon usage bias, while of 1.0 and >1.0 represent no bias and positive codon usage bias, respectively.

### Effective number of codons (ENC)

The ENc values were computed to assess the degree of codon usage bias in the polymerase coding sequences of EIV using the formula given below:

$$ ENc\kern0.5em =\kern0.5em 2\kern0.5em +\kern0.5em \frac{9}{{\overline{F}}_2}\kern0.5em +\kern0.5em \frac{1}{{\overline{F}}_3}\kern0.5em +\kern0.5em \frac{5}{{\overline{F}}_4}\kern0.5em +\kern0.5em \frac{3}{{\overline{F}}_6} $$

Where F_(*i* = 2,3,4,6)_ is the mean of F_i_ values for the i-fold degenerate amino acid. The F_i_ values were calculated using the formula given below:

$$ {\overline{F}}_i\kern0.5em =\kern0.5em \frac{n\underset{j\kern0.5em =\kern0.5em 1}{\overset{i}{\varSigma }}\kern0.5em {\left(\frac{nj}{n}\right)}^2-1}{n\kern0.5em -\kern0.5em 1} $$

Where n is the total number of occurrences of the codons for that amino acid and n_j_ is the total number of occurrences of the j^th^ codon for that amino acid. The ENc values range from 20 to 61 [95]. The ENc value of 20 states an extreme codon usage bias (only one of the possible synonymous codons is used for the corresponding amino acid), while that of 61 states no bias at all (all possible synonymous codons are used equally for the corresponding amino acid). Consequently, the smaller the ENc value, the greater will be the extent of codon usage bias. Altogether, a gene of ENc ≤ 35 is designated to possess strong codon bias [95, 96].

### ENc-plot

An ENc-plot is widely employed to see whether mutation bias/mutation pressure influence the codon usage bias or not. In this plot, the ENc values are the ordinate and the GC3s values (frequency of either a guanine or cytosine at the third codon position of the synonymous codons, excluding Met, Trp, and stop codons) are the abscissa [95]. If predicted ENc values lies on or around the standard curve (functional relation between expected ENc and GC3s), then codon usage is constrained only by G + C mutation bias. Else, other factors such as natural selection play significant role in shaping the codon usage bias. Expected ENc values were calculated as below:

$$ {ENc}_{expected}\kern0.5em =\kern0.5em 2\kern0.5em +\kern0.5em s\kern0.5em +\kern0.5em \frac{29}{s^2\kern0.5em +\kern0.5em \left(1\kern0.5em -\kern0.5em {s}^2\right)} $$

Where ‘s’ is the frequency of G + C at the third codon position of synonymous codons (i.e. GC3s).

### Parity rule 2 analysis

The Parity rule 2 (PR2) plot was constructed to estimate the effects of mutation pressure and natural selection on the codon usage of EIVs polymerase genes. In this plot, the AU-bias [A3/(A3 + U3)] at the third codon position of the four-codon amino acid of entire coding sequences is the ordinate and the GC-bias [G3/(G3 + C3)] is the abscissa. The center of the plot, where both coordinates crosses at 0.5, denotes no bias between the influence of the mutation pressure and natural selection [97, 98].

### Neutrality plot

A neutrality plot was generated where, GC12 is the ordinate and GC3 is abscissa. A plot regression with a slope of 0 (the points positioned on the parallel lines of the abscissa) indicates no effect of directional mutation pressure, while a slope of 1 (the points positioned on the diagonal line) is indicative of complete neutrality [99].

### General average hydropathicity (GRAVY) and aromaticity (AROMO)

The GRAVY values were calculated as a sum of the hydropathy values of all the amino acid in the gene product divided by the number of residues in the polymerase gene sequences [100]. These values range from −2 to 2; where positive and negative values are indicative of hydrophobic and hydrophilic proteins, respectively. AROMO value denotes the frequency of aromatic amino acid (Phe, Tyr, Trp). Both GRAVY and AROMO values are indices of amino acid usage, and the variation in amino acid compositions can also influence the results of codon usage analysis.

### Relative codon deoptimization index

The relative codon deoptimization index (RCDI) compares the similarity in codon usage of a given coding sequence with that of a reference genome. The RCDI values for the polymerase genes of EIV were computed using web-based RCDI/eRCDI server (http://genomes.urv.es/CAIcal/RCDI/). This server also calculates expected RCDI values for a set of sequences by generating random sequences with similar G + C content and amino acid compositions. The expected RCDI provides a direct threshold value for discerning whether the differences in the RCDI value are statistically significant and arise from the codon preferences or whether they are merely artifacts that arise from internal biases in the G + C composition and/or amino acid composition of the query sequences. The RCDI value of 1 specifies that the virus trails the host codon usage pattern and shows a host-adapted codon usage pattern. Contrariwise, RCDI values higher than 1 indicate the deoptimization of the codon usage patterns of the virus from that of its host(s) [101, 102].

### Similarity index

The similarity (SiD) index provides an insight into the influence of the overall codon usage pattern of the host on the formation of the overall codon usage of the virus, and was calculated as follows:

$$ {\displaystyle \begin{array}{l}R\left(A,\kern0.5em B\right)\kern0.5em =\kern0.5em \frac{\underset{i\kern0.5em =\kern0.5em 1}{\overset{59}{\varSigma }}{a}_i\kern0.5em \times \kern0.5em {b}_i}{\sqrt{\underset{i\kern0.5em =\kern0.5em 1}{\overset{59}{\varSigma }}\kern0.5em {a_i}^2\kern0.5em \times \kern0.5em \underset{i\kern0.5em =\kern0.5em 1}{\overset{59}{\varSigma }}\kern0.5em {b_i}^2}}\\ {}D\left(A,\kern0.5em B\right)\kern0.5em =\kern0.5em \frac{1\kern0.5em -\kern0.5em R\left(A,\kern0.5em B\right)}{2}\end{array}} $$

Where R(A,B) is defined as the cosine value of the angle included between the A and B spatial vectors, and represents the degree of similarity between the virus and host overall codon usage patterns. a_i_ is defined as the RSCU value for a specific codon among the 59 synonymous codons of the virus coding sequence. b_i_ is the RSCU value for the same codon in the host. D(A,B) represents the potential effect of the overall codon usage of the host on that of the virus, and its value range from 0 to 1.0 [103].

### Statistical analysis

Correspondence analysis (COA), a multivariate statistical method is widely used to study the trends in codon usage variations. In this analysis, the degrees of freedom were condensed to 40 (from 59 synonymous codons) by eliminating the variations caused by the unequal usage of amino acid while generating a correspondence analysis of RSCU [104]. The major trends within the dataset were estimated based on the measurement of relative inertia, and strains arranged according to their positions along the axes of major inertia. COA was performed on the RSCU values of codons and complete coding sequences of polymerase genes. In addition, Spearman’s rank correlation and linear regression analysis were executed by XLSTAT Version 2016 and GraphPad Prism 7.01 (GraphPad Software, San Diego, California, USA).

### Software and databases

Nucleotide compositions, relative synonymous codon usage (RSCU), GRAVY and AROMO, and correspondence analysis were calculated using the program CodonW 1.4.2 (by John Peden and available at http://sourceforge.net/projects/codonw/) [105]. The Effective Number of Codons (ENc) for each polymerase gene of EIV strains were computed using INCA2.1 [106]. Codon usage data of horse (*Equus caballus*), donkey (*Equus asinus*), and dog (*Canis familiaris*) was obtained from the codon usage database (available at: http://www.kazusa.or.jp/codon/) [107]. The RCDI was calculated using the approach of Puigbo et al. [80] (available at: http://genomes.urv.es/CAIcal/RCDI/). The frequencies of tRNAs in equine cells were retrieved from the GtRNAdb database [108].

## Additional files

Additional file 1: Table S1a. Consensus amino acid changes in the predicted PA protein compared to Richmond/1/07; **Table S1b.** Consensus amino acid changes in the predicted PA-X protein compared to Richmond/1/07; **Table S1c**. Consensus amino acid changes in the predicted PB1 protein compared to Richmond/1/07; **Table S1d.** Consensus amino acid changes in the predicted PB1-F2 protein compared to Richmond/1/07; **Table S1e.** Consensus amino acid changes in the predicted PB2 protein compared to Richmond/1/07. (DOC 742 kb)

Additional file 2: Figure S1. PA-X and PB1-F2 phylograms. (TIFF 2690 kb)

Additional file 3: Correlation coefficients among the position of genes along the first two major axes with various indexes of PA polymerase genes’ codon usage. (DOCX 33 kb)

Additional file 4: Figure S2. Comparison of Relative Synonymous Codon Usage (RSCU) patterns of EIVs polymerase genes with their host species. (TIFF 1828 kb)

Additional file 5: Figure S3. The similarity index analysis of of EIVs polymerase genes in relation to their host species, *Equus caballus. (TIFF 358 kb)*

Additional file 6: Table S6a. Correlation analysis among length (amino acid), GRAVY, AROMO, ENC, GC3s, ENc and the first two principle axes of COA of PB1 gene; **Table S6b.** Correlation analysis among length (amino acid), GRAVY, AROMO, ENC, GC3s, ENc and the first two principle axes of COA of PB2 gene; **Table S6c.** Correlation analysis among length (amino acid), GRAVY, AROMO, ENC, GC3s, ENc and the first two principle axes of COA of PA gene. (DOCX 18 kb)

Additional file 7: Primers used for amplification of polymerase genes of EIV. (DOCX 12 kb)

Additional file 8: Details of polymerase gene sequences of Equine Influenza virus (EIV) strains of equid origin used in the study. (DOC 69 kb)

## Abbreviations

A3s: A content on the third synonymous codon position
AROMO: Aromaticity
C3s: C content on the third synonymous codon position
COA: Correspondence analysis
EIV: Equine influenza virus
ENc: Effective number of codons
FEL: Fixed effect likelihood model
FUBAR: Fast unbiased bayesian approximation
G3s: G content on the third synonymous codon position
GARD: Genetic algorithm for recombination detection
GC1: GC content in the first position of the codons
GC12: Stands for the average value of GC content in the first and second position of the codons
GC2: GC content in the second position of the codons
GC3: GC content in the third position
GC3s: GC content on the third synonymous codon position
GRAVY: General average hydropathicity
MEME: Mixed effects model of evolution
PR2: Parity rule 2 analysis
RCDI: Relative codon deoptimization index
RSCU: Relative synonymous codon usage
SiD: Similarity Index
SLAC: Single likelihood ancestor counting
