# Supplementary material for: Genetic and codon usage bias analyses of polymerase genes of equine influenza virus and its relation to evolution
Source: BMC Genomics. 2017 Aug 23;18:652. doi: 10.1186/s12864-017-4063-1 (PMC5568313; doi:10.1186/s12864-017-4063-1)
Supplement: Supplementary file 1 — Consensus amino acid changes in the predicted PA protein compared to Richmond/1/07; Table S1b. Consensus amino acid changes in the predicted PA-X protein compared to Richmond/1/07; Table S1c. Consensus amino acid changes in the predicted PB1 protein compared to Richmond/1/07; Table S1d. Consensus amino acid changes in the predicted PB1-F2 protein compared to Richmond/1/07; Table S1e. Consensus amino acid changes in the predicted PB2 protein compared to Richmond/1/07. (DOC 742 kb) [file 12864_2017_4063_MOESM1_ESM.doc]

**Additional file 1a**. Consensus amino acid changes in the predicted PA protein compared to Richmond/1/07.

| **Strains** | **55** | **57** | **62** | **64** | **86** | **100** | **114** | **118** | **158** | **213** | **216** | **217** | **231** | **237** | **244** | **259** | **261** | **269** | **270** | **277** | **321** | **337** | **343** | **345** | **353** | **354** | **388** |
| --- | --- | --- | --- | --- | --- | --- | --- | --- | --- | --- | --- | --- | --- | --- | --- | --- | --- | --- | --- | --- | --- | --- | --- | --- | --- | --- | --- |
| Rich/07 | N | L | V | D | I | A | E | V | K | K | N | Y | V | K | S | P | S | K | M | H | N | T | E | I | R | I | S |
| Xinj/07 | . | . | . | . | . | . | K | . | R | . | . | . | . | . | . | . | . | . | . | . | . | . | . | . | . | . | . |
| Almi/07 | . | . | . | . | . | . | . | . | R | . | . | . | . | . | . | . | . | . | . | . | . | . | . | . | . | . | . |
| Athe/07 | - | - | - | - | - | - | - | - | R | . | . | . | . | . | . | . | . | . | . | . | . | . | . | . | . | . | . |
| Katr/08 | . | . | . | . | . | . | . | . | R | . | . | . | . | . | . | . | . | . | . | . | . | . | . | . | . | . | . |
| Myso/08 | . | . | . | . | . | . | . | . | R | . | . | . | . | . | . | . | . | . | . | . | . | . | . | . | . | . | . |
| Gans/08 | . | . | . | . | . | . | . | . | R | . | . | . | . | . | . | . | . | . | . | . | . | . | . | . | . | . | . |
| Hube/08 | . | . | . | . | . | . | K | . | R | . | . | . | . | . | . | . | . | . | . | . | . | . | . | . | . | . | . |
| Mong/08 | . | . | . | . | . | . | K | . | R | . | . | . | . | . | . | . | . | . | . | . | . | . | . | . | . | . | . |
| Ahme/09 | . | . | . | . | . | . | . | . | R | . | . | . | . | . | . | . | . | . | . | . | . | . | . | . | . | . | . |
| York/09 | . | . | . | . | . | . | . | . | R | . | . | . | . | . | . | . | . | . | . | . | . | . | . | . | . | . | . |
| Perth/09 | . | . | . | . | . | . | . | . | R | . | . | . | . | . | . | . | . | . | . | . | . | . | . | . | . | . | . |
| Heil/10 | . | . | . | . | . | . | K | . | R | . | . | . | . | . | . | . | . | . | . | . | . | . | . | . | . | . | . |
| Shro/10 | . | . | . | . | . | . | . | . | R | . | . | . | . | . | . | . | . | . | . | . | . | . | . | . | . | . | . |
| Devo/11 | . | . | . | . | . | . | . | . | R | . | . | . | . | . | . | . | . | . | . | . | . | . | . | . | . | . | . |
| E-Renf/11 | . | . | . | . | . | . | . | . | R | . | . | . | . | . | . | . | . | . | . | . | . | . | . | . | . | . | . |
| Xuzh/13 | . | . | . | . | . | . | K | . | G | . | . | . | . | . | . | . | . | . | . | . | . | . | . | . | . | . | . |
| N-Hamp/13 | . | . | . | . | . | . | . | . | R | . | . | . | . | . | . | . | . | . | . | . | . | . | . | . | . | . | . |
| Athen/03 | - | - | - | - | - | - | - | - | R | . | . | . | . | . | . | . | . | . | . | . | . | . | . | . | . | . | . |
| Newm/03 | . | . | . | E | M | . | . | . | . | . | . | . | . | E | . | . | . | . | . | . | S | A | . | . | . | . | . |
| Dub/12 | . | . | . | E | M | . | . | . | . | . | . | . | . | E | . | S | . | . | . | . | S | . | . | . | . | . | . |
| Kyon/11 | . | . | . | E | M | . | . | . | . | . | . | . | . | E | . | S | . | . | . | . | S | . | . | . | . | T | . |
| Kent/11 | . | . | . | E | M | . | . | . | . | . | . | . | . | E | . | S | . | . | . | . | S | . | . | . | . | . | . |
| Cali/10 | . | . | . | E | M | . | . | . | . | . | . | . | . | E | . | S | . | . | . | . | S | . | . | . | . | . | . |
| Lana/09 | . | . | . | E | M | . | . | . | . | . | . | . | . | E | . | S | . | . | . | . | S | . | . | . | . | . | . |
| Dors/09 | . | . | . | E | M | . | . | . | . | . | . | . | . | E | . | S | . | . | . | . | S | . | . | . | . | . | . |
| Linc/07 | . | . | . | E | M | . | . | . | . | . | . | . | . | E | . | S | . | . | . | . | S | . | . | . | . | . | . |
| Mont/07 | . | . | . | E | M | . | . | . | . | . | . | . | . | E | . | S | . | . | . | . | S | . | . | . | . | T | . |
| Ches/06 | . | . | . | E |  | . | . | . | . | . | . | . | . | E | . | . | . | . | . | . | S | A | A | L | K | T | N |
| Linc/06 | . | . | . | E | M | . | . | . | . | . | . | . | . | E | . | . | . | . | I | . | S | A | A | L | K | . | N |
| Ohio/05 | . | . | . | E | M | . | . | . | . | . | . | . | . | E | . | . | . | . | . | . | S | . | . | . | . | T | . |
| Wisc/03 | . | . | . | E | M | . | . | . | . | . | . | . | . | E | . | . | . | . | . | . | S | . | A | L | K | D | . |
| Kent/02 | . | . | . | E | M | . | . | . | . | . | . | . | . | E | . | . | . | . | . | . | S | . | . | . | . | T | . |
| Snai/98 | . | . | . | E | M | . | . | . | . | R | . | . | . | E | . | . | . | . | . | . | S | A | . | . | . | . | K |
| Kent/94 | . | . | I | E | M | . | . | . | . | R | . | . | . | E | . | . | . | . | . | . | S | A | A | L | K | T | N |
| Newm/1/93 | . | . | . | E | M | . | . | . | . | R | . | . | . | E | . | . | . | . | . | . | S | A | A | L | K | T |  |
| Newm/2/93 | . | . | I | E | M | . | . | . | . | R | . | . | . | E | . | . | . | . | I | . | S | A | A | L | K | T | N |
| Swit/93 | . | . | I | E | M | . | . | . | . | R | . | . | . | E | . | . | . | . | I | . | S | A | A | L | K | T | N |
| Kent/92 | . | . | I | E | M | . | . | . | . | R | . | . | . | E | . | . | . | . | . | . | S | A | A | L | K | T | N |
| Ital/92 | . | . | I | E | M | . | . | . | . | R | . | . | . | E | . | . | . | . | I | . | S | A | A | L | K | T | N |
| Kent/91 | . | . | I | E | M | . | . | . | . | R | . | . | . | E | . | . | . | . | . | . | S | A | A | L | K | T | N |
| Alas/91 | . | . | I | E | M | . | . | . | . | R | . | . | . | E | . | . | . | . | . | . | S | A | A | L | K | T | N |
| Kent/90 | . | . | I | E | M | . | . | . | . | R | . | . | . | E | . | . | . | . | I | . | S | A | A | L | K | T | N |
| Suss/89 | . | . | I | E | M | . | . | . | . | R | . | . | . | E | . | . | . | . | I | . | S | A | A | L | K | T | N |
| Kent/88 | . | . | I | E | M | . | . | . | . | R | . | . | . | E | . | . | . | . | I | . | S | A | A | L | K | T | N |
| Kent/87 | . | . | I | E | M | T | . | . | . | R | . | . | . | E | . | . | . | R | I | . | S | A | A | L | K | T | N |
| Joha/86 | . | . | I | E | M | . | . | . | . | R | . | . | A | E | . | . | . | R | I | . | S | A | A | L | K | T | . |
| Kent/86 | . | . | I | E | M | . | . | . | . | R | . | . | A | E | . | . | . | R | I | . | S | A | A | L | K | T | . |
| Sant/85 | . | . | I | E | M | . | . | . | . | R | . | . | A | E | . | . | . | R | I | . | S | A | A | L | K | T | . |
| Kent/81 | . | . | I | E | M | . | . | . | . | R | . | . | A | E | . | . | . | R | I | . | S | A | A | L | K | T | . |
| Kent/80 | . | . | I | E | M | . | . | . | . | R | . | . | A | E | . | . | . | R | I | . | S | A | A | L | K | T | . |
| Font/79 | . | . | I | E | M | . | . | . | . | R | . | . | A | E | . | . | . | R | I | . | S | A | A | L | K | . | . |
| Sau Pa/69 | D | R | I | E | M | I | . | I | . | R | D | H | A | E | G | L | . | R | I | S | . | A | A | L | K | . | . |
| Urug/63 | D | R | I | E | M | I | . | I | . | R | D | H | A | E | G | L | . | R | I | S | . | A | A | L | K | . | . |
| Miam/63 | D | . | I | E | M | V | . | I | . | R | D | H | A | E | G | L | . | R | I | S | . | A | A | L | K | . | . |

| **Strain** | **400** | **409** | **432** | **437** | **450** | **465** | **476** | **479** | **488** | **505** | **510** | **532** | **543** | **626** | **665** | **683** | **717** |
| --- | --- | --- | --- | --- | --- | --- | --- | --- | --- | --- | --- | --- | --- | --- | --- | --- | --- |
| Rich/07 | T | S | V | Y | I | I | T | E | K | I | H | F | L | R | K | I | * |
| Xing/07 | . | . | . | . | . | . | . | . | . | . | . | . | . | . | . | . | * |
| Almi/07 | . | . | . | . | . | . | . | . | . | . | . | . | . | . | . | . | * |
| Athe/07 | . | . | . | - | - | - | - | - | - | - | - | - | - | - | - | - | - |
| Katr/08 | . | . | . | . | . | . | . | . | . | . | . | . | . | . | . | . | * |
| Myso/08 | . | . | . | . | . | . | . | . | N | . | P | . | P | . | . | . | * |
| Gans/08 | . | . | . | . | . | . | . | . | . | . | . | . | . | . | . | . | * |
| Hube/08 | . | . | . | . | . | . | . | . | . | . | . | . | . | . | . | . | * |
| Mong/08 | . | . | . | . | . | . | . | . | . | . | . | . | . | . | . | . | * |
| Ahme/09 | . | . | . | . | . | . | . | . | N | . | P | . | P | . | . | . | * |
| York/09 | . | . | . | . | . | . | K | . | . | . | . | . | . | . | . | . | * |
| Perth/09 | . | . | . | . | . | . | . | . | . | . | . | . | . | . | . | . | * |
| Heil/10 | . | . | . | . | . | . | . | . | . | . | . | . | . | . | . | . | * |
| Shro/10 | . | . | . | . | . | . | . | . | . | . | . | . | . | . | . | . | * |
| Devo/11 | . | . | . | . | . | . | . | . | . | . | . | . | . | . | . | . | * |
| E-Renf/11 | . | . | . | . | . | . | . | . | . | . | . | . | . | . | . | . | * |
| Xuzh/13 | . | . | . | . | . | . | . | . | . | . | . | . | . | . | . | . | * |
| N-Hamp/13 | . | . | . | . | . | . | . | . | . | . | . | . | . | . | . | . | * |
| Athe/03 | . | . | . | - | - | - | - | - | - | - | - | - | - | - | - | - | - |
| Newm/03 | . | . | . | . | . | . | A | . | . | . | . | . | . | K | . | . | * |
| Dub/12 | . | N | . | . | . | V | A | . | . | V | . | . | . | K | . | . | * |
| Kyon/11 | . | N | . | . | . | V | A | . | . | V | . | . | . | K | . | . | * |
| Kent/11 | . | N | . | . | . | V | A | . | . | V | . | . | . | K | . | . | * |
| Cali/10 | . | N | . | . | . | V | A | . | . | V | . | . | . | K | . | . | * |
| Lana/09 | . | N | . | . | . | V | A | . | . | V | . | . | . | K | . | . | * |
| Dors/09 | . | N | . | . | . | V | A | . | . | V | . | . | . | K | . | . | * |
| Linc/1/07 | . | N | . | . | . | V | A | . | . | V | . | . | . | K | . | . | * |
| Mont/07 | . | N | . | . | . | V | A | . | . | V | . | . | . | K | . | . | * |
| Cesh/06 | . | . | . | . | V | . | A | . | . | . | . | . | . | K | . | . | * |
| Linc/06 | . | . | I | . | V | . | A | . | . | . | . | . | . | K | . | . | * |
| Ohio/05 | . | . | . | . | . | V | A | . | . | V | . | . | . | K | . | . | * |
| Wisc/03 | . | . | . | . | V | . | A | . | . | V | . | . | . | K | . | . | * |
| Kent/02 | . | . | . | . | . | . | A | . | . | . | . | . | . | K | . | . | * |
| Snail/98 | . | . | . | . | . | . | A | . | . | . | . | . | . | K | . | . | * |
| Kent/94 | . | . | I | . | V | . | A | . | . | . | . | . | . | K | . | . | * |
| Newm/1/93 | . | . | . | . | . | . | A | . | . | . | . | . | . | K | . | . | * |
| Newm/2/93 | . | . | I | . | F | . | A | . | . | . | . | . | . | K | . | . | * |
| Swit/93 | . | . | I | . | V | . | A | . | . | . | . | . | . | K | . | . | * |
| Kent/92 | . | . | . | . | V | . | A | . | . | . | . | . | . | K | . | . | * |
| Ital/92 | . | . | I | . | V | . | A | . | . | . | . | . | . | K | . | . | * |
| Kent/91 | . | . | I | . | V | . | A | . | . | . | . | . | . | K | . | . | * |
| Kent/90 | . | . | I | . | V | . | A | . | . | . | . | . | . | K | . | . | * |
| Suss/89 | . | . | I | . | V | . | A | . | . | . | . | . | . | K | . | . | * |
| Kent/88 | . | . | I | . | V | . | A | . | . | . | . | . | . | K | . | . | * |
| Kent/87 | . | . | I | . | V | . | A | . | . | . | . | . | . | K | . | . | * |
| Joha/86 | . | . | I | . | V | . | A | . | . | . | . | L | . | K | . | . | * |
| Kent/86 | . | . | I | . | V | . | A | . | . | . | . | L | . | K | . | . | * |
| Sant/85 | . | . | I | . | V | . | A | . | . | . | . | L | . | K | . | . | * |
| Kent/81 | . | . | I | . | V | . | A | D | . | . | . | L | L | K | . | . | * |
| Kent/80 | . | . | I | . | V | . | A | D | . | . | . | L | . | K | . | . | * |
| Newm/79 | . | . | I | . | V | . | A | D | . | . | . | L | . | K | . | . | * |
| Font/79 | . | . | I | . | V | . | A | D | . | . | . | L | L | K | . | . | * |
| Sau Pa/69 | P | . | . | H | V | . | A | D | . | . | . | L | . | K | I | L | * |
| Urug/63 | P | . | . | H | V | . | A | D | . | . | . | L | . | K | I | L | * |
| Miam/63 | A | . | . | H | V | . | A | D | . | . | . | L | L | K | I | L | * |

**Additional file 1a. Consensus amino acid changes in the predicted PA protein compared to Richmond/1/07.**

**Additional file 1b. Consensus amino acid changes in the predicted PA-X protein compared to Richmond/1/07**

| **Strains** | **62** | **64** | **86** | **100** | **114** | **118** | **158** | **210** | **213** | **216** | **228** | **231** | **240** | **244** | **253** |
| --- | --- | --- | --- | --- | --- | --- | --- | --- | --- | --- | --- | --- | --- | --- | --- |
| Rich/07 | V | D | I | A | E | V | K | * | S | I | I | S | A | V | * |
| Xinj/07 | . | . | . | . | K | . | R | R | . | . | . | . | . | . | * |
| Almi/07 | . | . | . | . | . | . | R | R | . | . | . | . | . | . | * |
| Katr/08 | . | . | . | . | . | . | R | R | . | . | . | . | . | . | * |
| Myso/08 | . | . | . | . | . | . | R | R | . | . | . | . | . | . | * |
| Gans/08 | . | . | . | . | . | . | R | R | . | . | . | . | . | . | * |
| Hube/08 | . | . | . | . | K | . | R | R | . | . | . | . | . | . | * |
| Mong/08 | . | . | . | . | K | . | R | R | . | . | . | . | . | . | * |
| Ahme/09 | . | . | . | . | . | . | R | R | . | . | . | . | . | . | * |
| York/09 | . | . | . | . | . | . | R | R | . | . | . | . | . | . | * |
| Perth/09 | . | . | . | . | . | . | R | R | . | . | . | . | . | . | * |
| Heil/10 | . | . | . | . | K | . | R | R | . | . | . | . | . | . | * |
| Shro/10 | . | . | . | . | . | . | R | R | . | . | . | . | . | . | * |
| Devo/11 | . | . | . | . | . | . | R | R | . | . | . | . | . | . | * |
| E-Renf/11 | . | . | . | . | . | . | R | R | . | . | . | . | . | . | * |
| Xuzh/13 | . | . | . | . | K | . | G | R | . | . | . | . | . | . | * |
| N-Hamp/13 | . | . | . | . | . | . | R | R | . | . | . | . | . | . | * |
| Newm/03 | . | E | M | . | . | . | . | R | . | . | . | . | D | . | * |
| Dub/12 | . | E | M | . | . | . | . | R | . | . | . | . | D | . | * |
| Kyon/11 | . | E | M | . | . | . | . | R | . | . | . | . | . | . | * |
| Kent/11 | . | E | M | . | . | . | . | R | . | . | . | . | D | . | * |
| Cali/10 | . | E | M | . | . | . | . | R | . | . | . | . | . | . | * |
| Lana/09 | . | E | M | . | . | . | . | R | . | . | . | . | D | . | * |
| Dors/09 | . | E | M | . | . | . | . | R | . | . | . | . | D | . | * |
| Linc/07 | . | E | M | . | . | . | . | R | . | . | . | . | N | . | * |
| Mont/07 | . | E | M | . | . | . | . | R | . | . | . | . | . | . | * |
| Ches/06 | . | E | . | . | . | . | . | R | G | . | . | . | . | . | * |
| Linc/06 | . | E | M | . | . | . | . | Q | G | . | . | . | . | . | * |
| Ohio/05 | . | E | M | . | . | . | . | R | . | . | . | . | . | . | * |
| Wisc/03 | . | E | M | . | . | . | . | R | . | . | . | . | . | . | * |
| Kent/02 | . | E | M | . | . | . | . | R | . | . | . | . | . | . | * |
| Snai/98 | . | E | M | . | . | . | . | R | G | . | . | . | . | . | * |
| Kent/94 | I | E | M | . | . | . | . | R | G | . | . | . | . | . | * |
| Newm/1/93 | . | E | M | . | . | . | . | R | G | . | . | . | . | . | * |
| Newm/2/93 | I | E | M | . | . | . | . | R | G | . | . | . | . | . | * |
| Swit/93 | I | E | M | . | . | . | . | R | G | . | . | . | . | . | * |
| Kent/92 | I | E | M | . | . | . | . | R | G | . | . | . | . | . | * |
| Ital/92 | I | E | M | . | . | . | . | R | G | . | . | . | . | . | * |
| Kent/91 | I | E | M | . | . | . | . | R | G | . | . | . | . | . | * |
| Alas/91 | I | E | M | . | . | . | . | R | G | . | . | . | . | . | * |
| Kent/90 | I | E | M | . | . | . | . | R | G | . | . | . | . | . | * |
| Suss/89 | I | E | M | . | . | . | . | R | G | . | . | . | . | . | * |
| Kent/88 | I | E | M | . | . | . | . | R | G | . | . | . | . | . | * |
| Kent/87 | I | E | M | T | . | . | . | R | G | . | . | . | . | . | * |
| Joha/86 | I | E | M | . | . | . | . | Q | G | . | . | P | . | A | * |
| Kent/86 | I | E | M | . | . | . | . | Q | G | . | . | P | . | A | * |
| Sant/85 | I | E | M | . | . | . | . | Q | G | . | . | P | . | A | * |
| Kent/81 | I | E | M | . | . | . | . | Q | G | . | . | P | . | A | * |
| Kent/80 | I | E | M | . | . | . | . | Q | G | . | . | P | . | A | * |
| Font/79 | I | E | M | . | . | . | . | Q | G | . | . | P | . | A | * |
| Sau Pa/69 | I | E | M | I | . | I | . | Q | G | T | T | P | . | A | * |
| Urug/63 | I | E | M | V | . | I | . | Q | G | T | T | P | . | A | * |
| Miam/63 | I | E | M | V | . | I | . | Q | G | T | T | P | . | A | * |

M2 Protein

**Additional file 1c**. Consensus amino acid changes in the predicted PB1 protein compared to Richmond/1/07

| **Strains** | **61** | **94** | **114** | **119** | **154** | **164** | **198** | **221** | **275** | **317** | **329** | **377** | **397** | **457** | **584** | **587** | **618** | **621** | **738** | **758** |
| --- | --- | --- | --- | --- | --- | --- | --- | --- | --- | --- | --- | --- | --- | --- | --- | --- | --- | --- | --- | --- |
| Rich/1/07 | I | F | I | M | G | M | R | T | G | I | R | E | V | E | R | T | D | K | D | * |
| Xinj/07 | . | . | . | . | . | . | . | . | . | . | . | . | . | G | . | . | . | . | . | * |
| Almi/07 | . | . | . | . | . | . | . | . | - | - | - | - | - | - | - | - | - | - | - | - |
| Athe/07 | - | - | . | . | . | . | . | . | . | . | Q | D | . | . | - | - | - | - | - | - |
| Katr/08 | . | . | . | . | . | . | . | . | . | . | . | . | . | G | . | . | . | . | . | * |
| Myso/08 | . | . | . | . | . | . | . | . | V | . | . | . | . | G | . | . | . | . | . | * |
| Gans/08 | . | . | . | . | . | . | . | . | . | . | . | . | . | G | . | . | . | . | . | * |
| Hube/08 | . | . | . | . | . | . | . | . | . | . | . | . | . | G | . | . | . | . | . | * |
| Mong/08 | . | . | . | . | . | . | . | . | . | . | . | . | . | G | - | - | - | - | - | - |
| Ahme/09 | . | . | . | . | . | . | . | . | V | . | . | . | . | G | . | . | . | . | . | * |
| York/09 | . | . | . | . | . | . | . | . | . | . | . | . | . | . | . | . | . | . | . | * |
| Perth/09 | . | L | . | V | . | . | . | . | . | . | Q | D | . | . | . | . | E | R | . | * |
| Heil/10 | . | . | . | . | . | . | . | . | . | . | . | . | . | G | . | . | . | . | . | * |
| Shro/10 | . | . | . | . | . | . | . | A | . | . | . | . | . | . | . | . | . | . | . | * |
| Devo/11 | . | . | . | . | . | . | . | A | . | . | . | . | . | . | . | . | . | . | . | * |
| E-Renf/11 | . | . | . | . | . | . | . | A | . | . | . | . | . | . | . | . | . | . | . | * |
| Xuzh/13 | . | . | . | . | . | . | . | . | . | . | . | . | . | G | . | . | . | . | . | * |
| N-Hamp/13 | . | . | . | . | . | . | . | A | . | . | . | . | . | . | . | . | . | . | . | * |
| Athe/03 | - | - | . | . | . | . | . | . | . | . | Q | D | . | . | - | - | - | - | - | - |
| Newm/03 | . | . | . | V | . | . | . | . | . | . | Q | D | . | . | . | . | E | . | . | * |
| Duba/12 | . | L | . | V | . | . | . | . | . | . | Q | D | . | . | Q | . | E | R | . | * |
| Kyon/11 | . | L | . | V | . | . | . | . | . | . | Q | D | . | . | . | . | E | R | . | * |
| Kent/11 | . | L | . | V | . | . | . | . | . | . | Q | D | . | . | Q | . | E | R | . | * |
| Cali/10 | . | L | . | V | . | . | . | . | . | . | Q | D | . | . | Q | . | E | R | . | * |
| Lana/09 | . | L | . | V | . | . | . | . | . | . | Q | D | . | . | . | . | E | R | . | * |
| Dors/09 | . | L | . | V | . | . | . | . | . | . | Q | D | . | . | . | . | E | R | . | * |
| Linc/07 | . | L | . | V | . | . | . | . | . | . | Q | D | . | . | . | . | E | R | . | * |
| Mont/07 | . | L | . | V | . | . | . | . | . | . | Q | D | . | . | . | . | E | R | . | * |
| Ches/06 | . | . | V | V | D | . | . | A | . | . | Q | D | . | . | . | . | E | . | . | * |
| Linc/06 | . | . | V | V | D | . | . | A | . | . | Q | D | . | . | . | . | E | . | E | * |
| Ohio/05 | . | . | . | V | . | . | . | . | . | . | Q | D | . | . | . | . | E | R | . | * |
| Wis/03 | . | . | . | V | . | . | . | . | . | . | Q | D | . | . | . | . | E | . | . | * |
| Kent/02 | . | . | . | V | . | . | . | . | . | . | Q | D | . | . | . | . | E | . | . | * |
| Snai/98 | . | . | V | V | D | . | . | A | . | M | Q | D | . | . | . | . | E | . | E | * |
| Kent/94 | . | . | V | V | D | . | . | A | . | . | Q | D | . | . | . | . | E | . | . | * |
| Newm/1/93 | . | . | V | V | D | . | . | A | . | . | Q | D | . | . | . | . | E | . | E | * |
| Newm/2/93 | . | . | V | V | D | . | . | A | . | M | Q | D | . | . | . | . | E | . | E | * |
| Swit/93 | . | . | V | V | D | . | . | A | . | M | Q | D | . | . | . | . | E | . | E | * |
| Kent/92 | . | . | V | V | D | . | . | A | . | M | Q | D | . | . | . | . | E | . | . | * |
| Ital/92 | . | . | V | V | D | . | . | A | . | M | Q | D | . | . | . | . | E | . | E | * |
| Kent/91 | . | . | V | V | D | . | . | A | . | M | Q | D | . | . | . | . | E | . | . | * |
| Alas/91 | . | . | V | V | D | . | . | A | . | M | Q | D | . | . | . | . | E | . | . | * |
| Kent/90 | . | . | V | V | D | . | . | A | . | M | Q | D | . | . | . | . | E | . | . | * |
| Suss/89 | . | . | V | V | D | . | . | A | . | M | Q | D | . | . | . | . | E | . | E | * |
| Kent/88 | . | . | V | V | D | . | . | A | . | M | Q | D | . | . | . | . | E | . | E | * |
| Kent/87 | . | . | V | V | D | . | K | A | . | M | Q | D | . | . | . | . | E | . | E | * |
| Joha/86 | . | . | V | V | D | . | K | A | . | M | Q | D | . | . | . | . | E | . | E | * |
| Kent/86 | . | . | V | V | D | . | K | A | . | M | Q | D | . | . | . | . | E | . | E | * |
| Sant/85 | . | . | V | V | D | . | K | A | . | M | Q | D | I | . | . | . | E | . | E | * |
| Kent/81 | . | . | V | V | D | . | K | A | . | M | Q | D | I | . | . | . | E | . | E | * |
| Kent/80 | . | . | V | V | D | . | K | A | . | M | Q | D | I | . | . | . | E | . | E | * |
| Newm/79 | . | . | V | V | D | . | K | A | . | M | Q | D | I | . | Q | . | E | . | E | * |
| Font/79 | . | . | V | V | D | . | K | A | . | M | Q | D | I | . | Q | . | E | . | E | * |
| Saop/69 | T | . | V | V | D | I | K | A | . | M | Q | D | I | . | K | A | E | Q | E | * |
| Urug/63 | T | . | V | V | D | I | K | A | . | M | Q | D | I | . | . | A | E | Q | E | * |
| Miam/63 | T | . | V | V | D | I | K | A | . | M | Q | D | I | . | . | A | E | Q | E | * |

**Additional file 1d**. Consensus amino acid changes in the predicted PB1-F2 protein compared to Richmond/1/07

| **Strains** | **4** | **16** | **18** | **20** | **21** | **35** | **40** | **41** | **42** | **49** | **50** | **63** | **66** | **68** | **70** | **74** | **77** | **79** | **80** | **82**  **1** | **83** | **84** | **85** | **86** | **87** | **88** | **89** | **90** | **91** |
| --- | --- | --- | --- | --- | --- | --- | --- | --- | --- | --- | --- | --- | --- | --- | --- | --- | --- | --- | --- | --- | --- | --- | --- | --- | --- | --- | --- | --- | --- |
| Rich/1/07 | E | T | I | K | R | L | D | H | F | V | D | S | N | I | E | I | L | Q | W | * | - | - | - | - | - | - | - | - | - |
| Xinj/07 | . | . | . | . | . | . | . | . | . | . | G | . | . | . | . | . | . | . | . | * | - | - | - | - | - | - | - | - | - |
| Almi/07 | - | . | . | . | . | . | . | . | . | . | G | . | . | . | . | . | . | . | . | * | - | - | - | - | - | - | - | - | - |
| Katr/08 | . | . | . | . | . | . | . | . | S | . | G | . | . | . | . | . | . | . | . | * | - | - | - | - | - | - | - | - | - |
| Mys/08 | . | . | . | . | . | . | . | . | S | . | G | . | . | . | . | . | . | . | . | * | - | - | - | - | - | - | - | - | - |
| Gans/08 | . | . | . | . | . | . | . | . | . | . | G | . | . | . | . | . | . | . | . | * | - | - | - | - | - | - | - | - | - |
| Hube/08 | . | . | . | . | . | . | . | . | . | . | G | . | . | . | . | . | . | . | . | * | - | - | - | - | - | - | - | - | - |
| Mong/08 | . | . | . | . | . | . | . | . | . | . | G | . | . | . | . | . | . | . | . | * | - | - | - | - | - | - | - | - | - |
| Ahm/09 | . | . | . | . | . | . | . | . | S | . | G | . | . | . | . | . | . | . | . | * | - | - | - | - | - | - | - | - | - |
| York/09 | . | . | . | . | . | . | . | . | . | . | G | . | . | . | . | . | . | . | . | * | - | - | - | - | - | - | - | - | - |
| Perth/09 | G | . | . | . | K | . | . | . | . | . | G | Y | . | . | . | . | . | R | . | * | - | - | - | - | - | - | - | - | - |
| Heil/10 | . | . | . | . | . | . | . | . | . | . | G | . | . | . | . | . | . | . | . | * | - | - | - | - | - | - | - | - | - |
| Shro/10 | . | . | . | . | . | . | . | . | . | . | V | . | . | . | . | . | . | . | . | * | - | - | - | - | - | - | - | - | - |
| Devo/11 | . | . | . | . | . | . | . | . | . | . | V | . | . | . | . | . | . | . | . | * | - | - | - | - | - | - | - | - | - |
| E-Renf/11 | . | . | . | . | . | . | . | . | . | . | V | . | . | . | . | . | . | . | . | * | - | - | - | - | - | - | - | - | - |
| Xuz/13 | . | . | . | . | . | . | . | . | . | . | G | . | . | . | . | . | . | . | . | * | - | - | - | - | - | - | - | - | - |
| N-Hamp/13 | . | . | . | . | . | . | . | . | . | . | V | . | . | . | . | . | . | . | . | * | - | - | - | - | - | - | - | - | - |
| New/03 | G | . | . | . | . | . | . | . | . | . | G | . | . | . | . | . | . | R | . | * | - | - | - | - | - | - | - | - | - |
| Duba/12 | G | . | . | . | K | . | . | . | . | . | . | Y | . | . | . | . | . | R | . | * | - | - | - | - | - | - | - | - | - |
| Kyon/11 | G | . | . | . | K | . | . | . | . | . | G | Y | . | . | G | . | . | R | . | * | - | - | - | - | - | - | - | - | - |
| Kent/11 | G | . | . | . | K | . | . | . | . | . | . | Y | . | . | . | . | . | R | . | * | - | - | - | - | - | - | - | - | - |
| Cali/10 | G | . | . | . | K | . | . | . | . | . | G | Y | . | . | . | . | . | R | . | * | - | - | - | - | - | - | - | - | - |
| Lana/09 | G | . | . | . | K | . | . | . | . | . | G | Y | . | . | . | . | . | R | . | * | - | - | - | - | - | - | - | - | - |
| Dors/09 | G | . | . | . | K | . | . | . | . | . | G | Y | . | . | . | . | . | R | . | * | - | - | - | - | - | - | - | - | - |
| Linc/1/07 | G | . | . | . | K | . | . | . | . | . | G | Y | . | . | . | . | . | R | . | * | - | - | - | - | - | - | - | - | - |
| Mont/07 | G | . | . | . | K | . | . | . | . | . | G | Y | . | . | . | . | . | R | . | * | - | - | - | - | - | - | - | - | - |
| Ches/06 | G | . | T | . | . | . | . | R | . | . | G | . | . | . | . | . | . | R | . | W | F | S | R | Q | E | W | T | N | * |
| Linc/06 | G | I | T | . | . | . | . | R | . | A | V | . | . | . | G | . | . | R | * | - | - | - | - | - | - | - | - | - | - |
| Ohi/05 | G | . | . | . | K | . | . | . | . | . | G | Y | . | . | . | . | . | R | . | * | - | - | - | - | - | - | - | - | - |
| Ohio/03 | G | . | . | . | K | . | . | . | . | . | G | . | . | . | . | . | . | R | . | * | - | - | - | - | - | - | - | - | - |
| Wisc/03 | G | . | . | . | K | . | . | . | . | . | G | . | . | . | . | . | . | R | . | * | - | - | - | - | - | - | - | - | - |
| Ken/02 | G | . | . | . | . | . | . | . | . | . | G | . | . | . | . | . | . | R | . | * | - | - | - | - | - | - | - | - | - |
| Snai/98 | G | I | T | . | . | . | . | R | . | A | V | . | . | . | G | . | . | R | * | - | - | - | - | - | - | - | - | - | - |
| Newm/1/93 | G | . | . | . | . | . | . | R | . | . | G | . | . | . | . | . | . | R | . | W | F | S | R | Q | E | W | T | N | * |
| Newm/2/93 | G | I | T | . | . | . | . | R | . | A | V | . | . | . | G | . | . | R | . | W | F | S | R | Q | E | W | T | N | * |
| Swit/93 | G | I | T | . | . | . | . | R | . | A | V | . | . | . | G | . | . | R | . | W | F | S | R | Q | E | W | T | N | * |
| Kent/92 | G | I | T | . | . | . | . | R | . | . | G | . | . | . | . | . | . | . | . | W | F | S | R | Q | E | W | T | N | * |
| Ital/92 | G | I | T | . | . | . | . | R | . | A | G | . | . | . | G | . | . | R | . | W | F | S | R | Q | E | W | T | N | * |
| Kent/91 | G | I | T | . | . | . | . | R | . | . | G | . | . | . | . | . | . | . | . | W | F | S | R | Q | E | W | T | N | * |
| Alas/91 | G |  | T | . | . | . | . | R | . | . | G | . | . | . | . | . | . | R | . | W | F | S | R | Q | E | W | T | N | * |
| Kent/90 | G |  | T | . | . | . | . | R | . | . | G | . | . | . | . | . | . | R | . | W | F | S | R | Q | E | W | T | N | * |
| Suss/89 | GG |  | T | . | . | . | . | R | . | A | G | . | . | . | . | . | . | R | . | W | F | S | R | Q | E | W | T | N | * |
| Kent/88 | G |  | T |  |  |  |  | R |  | A | G |  |  |  |  |  |  | R |  | W | F | S | R | Q | E | W | T | N | * |
| Kent/87 | G | . | T | . | . | . | . | R |  | A | G | . | . | . | . | . | . | R | . | W | F | S | R | Q | E | W | T | N | * |
| Joha/86 | G | . | T | . | . | . | . | R |  | A | G | . | . | . | . | . | . | R | . | W | F | S | R | Q | E | W | T | N | * |
| Kent/86 | G | . | T | . | . | S | . | R |  | A | G | . | . | . | . | . | . | R | . | W | F | S | R | Q | E | W | T | N | * |
| Sant/85 | G | . | T | . | . | S | . | R |  | A | . | . | . | . | G | . | . | R | . | W | F | S | R | Q | E | W | T | N | * |
| Kent/81 | G | . | T | . | . | S | . | R | C | A | . | . | . | . | G | . | . | R | . | W | F | S | R | Q | E | W | T | N | * |
| Kent/80 | G | . | T | . | . | S | . | R | C | A | . | . | . | . | G | . | . | R | . | W | F | S | R | Q | E | W | T | N | * |
| Newm/79 | G | . | T | . | . | S | . | R | C | A | . | . | S | . | G | . | . | R | . | W | F | S | R | Q | E | W | T | N | * |
| Font/79 | G | . | T | . | E | S | . | R | C | A | . | . | S | . | G | . | . | R | . | W | F | S | R | Q | E | W | T | N | * |
| SaoP/69 | . | . | T | R | . | S | G | R | C | A | . | . | S | T | G | T | S | R | . | W | F | S | R | Q | E | W | T | N | * |
| Urug/63 | . | . | T | R | . | S | G |  | C | A | . | . | S | T | G | T | S | R | . | W | F | S | R | Q | E | W | T | N | * |
| Miam/63 | . | . | T | R | . | S | G | R | C | A | . | . | S | T | G | T | S | R | . | W | F | S | R | Q | E | W | T | N | * |

**Additional file 1e. Consensus amino acid changes in the predicted PB2 protein compared to A/equine/**Richmond/1/07

| Strains | **12** | **65** | **105** | **107** | **109** | **147** | **251** | **299** | **344** | **380** | **398** | **511** | **575** | **582** | **588** | **590** | **606** | **613** | **633** | **660** | **667** | **684** | **686** | **715** | **717** | **760** |
| --- | --- | --- | --- | --- | --- | --- | --- | --- | --- | --- | --- | --- | --- | --- | --- | --- | --- | --- | --- | --- | --- | --- | --- | --- | --- | --- |
| Rich/1/07 | L | E | A | S | I | V | K | K | M | R | I | I | I | S | T | S | V | A | F | K | V | A | V | K | A | * |
| Xinj/07 | . | . | . | . | . | . | . | . | . | . | . | . | . | . | . | . | . | . | . | . | . | . | . | . | . | * |
| Athe/07 | - | - | - | . | . | . | . | . | . | . | . | - | - | - | - | - | - | - | - | - | - | - | - | - | - | - |
| Katr/08 | . | . | . | . | . | . | . | . | . | K | . | . | . | . | . | . | I | . | . | . | . | . | . | . | . | * |
| Myso/08 | . | . | . | . | . | . | . | . | . | K | . | . | . | F | . | . | I | . | S | . | . | . | . | . | . | * |
| Gans/08 | . | . | . | . | . | . | . | . | . | . | . | . | . | . | . | . | . | . | . | . | . | . | . | . | . | * |
| Hube/08 | . | . | . | . | . | . | . | . | . | . | . | . | . | . | . | . | . | . | . | . | . | . | . | . | . | * |
| Mong/08 | . | . | . | . | . | . | . | . | . | K | . | . | . | . | . | . | . | . | . | . | . | . | . | . | . | * |
| Ahm/09 | . | . | . | . | . | . | . | . | . | K | . | . | . | F | . | . | I | . | S | . | . | . | . | . | . | * |
| York/09 | . | . | . | N | . | . | . | . | . | . | . | . | . | . | . | . | . | . | . | R | . | . | . | . | . | * |
| Perth/09 | . | . | . | N | . | . | . | . | . | . | . | . | . | . | . | . | . | . | . | . | . | . | . | . | . | * |
| Heil/10 | . | . | . | . | . | . | . | . | . | . | . | . | . | . | . | . | . | . | . | . | . | . | . | . | . | * |
| Shro/10 | . | . | . | N | . | . | . | . | . | . | . | . | . | . | . | . | . | . | . | . | . | . | . | . | . | * |
| Devo/11 | . | . | . | N | . | . | . | . | . | . | . | . | . | . | . | . | . | . | . | . | . | . | . | . | . | * |
| E-Renf/11 | . | . | . | N | . | . | . | . | . | . | . | . | . | . | . | . | . | . | . | . | . | . | . | . | . | * |
| Xuzh/13 | . | . | . | . | . | . | . | . | . | . | . | . | . | . | . | . | . | . | . | . | . | . | . | . | . | * |
| N-Hamp/13 | . | . | . | N | . | . | . | . | . | . | . | . | . | . | . | . | . | . | . | . | . | . | . | . | . | * |
| Athe/03 | - | - | - | . | . | . | . | . | . | . | . | - | - | - | - | - | - | - | - | - | - | - | - | - | - | - |
| Newm/03 | . | . | T |  |  |  | R | . | . | . | . | . | . | . | . | . | . | . | . | . | . | . | . | . | . | * |
| Duba/12 | . | . | T | . | . | . | R | . | . | . | V | . | . | . | . | . | . | . | . | R | I | T | I | . | . | * |
| Kyon/11 | . | . | T | . | . | . | R | . | . | . | . | . | . | . | . | . | . | . | . | . | . | . | . | . | . | * |
| Kent/11 | . | . | T | . | . | . | R | . | . | . | V | . | . | . | . | . | . | . | . | R | I | T | I | . | . | * |
| Cali/10 | . | . | T | . | . | . | R | . | . | . | . | . | . | . | . | . | . | . | . | R | I | T | I | . | . | * |
| Lana/09 | . | . | . | N | . | . | . | . | . | . | . | . | . | . | . | . | . | . | . | . | . | . | . | . | . | * |
| Dors/09 | . | . | P | N | . | . | . | . | . | . | . | . | . | . | . | . | . | . | . | . | . | . | . | . | . | * |
| Linc/07 | . | . | T | . | . | . | R | . | . | . | V | . | . | . | . | . | . | . | . | R | . | . | . | . | . | * |
| Mont/07 | . | . | T | . | . | . | R | . | . | . | V | . | . | . | . | . | . | . | . | R | . | . | . | . | . | * |
| Cesh/06 | S | . | T | . | . | . | . | . | . | . | . | . | . | . | . | . | . | . | . | . | . | . | . | . | . | * |
| Linc/06 | S | . | T | . | . | . | . | . | . | . | . | . | . | . | . | . | . | . | . | . | . | . | . | . | T | * |
| Tot/07 | . | . | T | . | . | . | R | . | . | . | . | . | . | . | . | . | . | . | . | . | . | . | . | . | . | * |
| Ohi/05 | . | . | T | . | . | . | R | . | . | . | V | . | . | . | . | . | . | . | . | . | . | . | . | . | . | * |
| Wis/03 | . | . | T | . | . | . | R | . | . | . | . | . | . | . | . | . | . | . | . | . | . | . | . | . | . | * |
| Ken/02 | . | . | T | . | . | . | R | . | . | . | . | . | . | . | . | . | . | . | . | . | . | . | . | . | . | * |
| Snail/98 | S | . | T | . | . | . | . | . | . | . | V | . | . | . | . | . | . | . | . | . | . | . | . | . | . | * |
| Kent/94 | S | . | T | . | . | . | R | . | . | . | . | . | . | . | . | . | . | . | . | . | . | . | . | . | . | * |
| New/1/93 | . | . | T | . | . | . | R | . | . | . | . | . | . | . | . | . | . | . | . | . | . | . | . | . | T | * |
| New/2/93 | S | . | T | . | . | . | R | . | . | . | . | . | . | . | . | . | . | . | . | . | . | . | . | . | T | * |
| Swit/93 | S | . | T | . | . | . | R | . | . | . | . | . | . | . | . | . | . | . | . | . | . | . | . | . | T | * |
| Kent/92 | S | . | T | . | . | . | R | . | . | . | . | . | . | . | . | . | . | . | . | . | . | . | . | . | . | * |
| Ital/92 | S | . | T | . | . | . | R | . | . | . | . | . | . | . | . | . | . | . | . | . | . | . | . | . | T | * |
| Kent/91 | S | . | T | . | . | . | R | . | . | . | . | . | . | . | . | . | . | . | . | . | . | . | . | . | . | * |
| Alas/91 | S | . |  | . | . | . | . | . | . | . | . | . | . | . | . | . | . | . | . | . | . | . | . | . | . | * |
| Kent/90 | S | . | T | . | . | . | R | . | . | . | . | . | . | . | . | . | . | . | . | . | . | . | . | . | . | * |
| Suss/89 | S | . | T | . | . | . | R | . | . | . | . | . | . | . | . | . | . | . | . | . | . | . | . | . | T | * |
| Kent/88 | S | . | T | . | . | . | R | . | . | . | . | . | . | . | . | . | . | . | . | . | . | . | . | . | T | * |
| Kent/87 | S | . | T | . | . | . | R | . | . | . | . | . | . | . | . | . | . | . | . | . | . | . | . | . | T | * |
| Joha/86 | S | . | T | . | . | . | R | . | . | . | . | . | . | . | . | . | . | . | . | . | . | . | . | . | T | * |
| Kent/86 | S | . | T | . | . | . | R | . | . | . | . | . | . | . | . | . | . | . | . | . | . | . | . | . | T | * |
| Sant/85 | S | . | T | . | V | . | R | . | . | . | . | . | . | . | . | . | . | . | . | . | . | . | . | . | T | * |
| Ken/81 | S | . | T | . | . | . | R | . | V | . | . | V | . | . | . | G | . | . | . | . | . | . | . | . | T | * |
| Kent/80 | S | . | T | . | . | . | R | . | V | . | . | V | . | . | . | G | . | . | . | . | . | . | . | . | T | * |
| Font/79 | S | . | T | . | . | . | R | . | V | . | . | V | . | . | . | G | . | . | . | . | . | . | . | . | T | * |
| SaoP/69 | S | G | T | . | V | I | R | R | V | . | . | V | M | . | A | G | . | V | . | . | . | . | . | N | . | * |
| Urug/63 | S | G | T | . | V | I | R | R | V | . | . | V | M | . | A | G | . | V | . | . | . | . | . | N | . | * |
| Mia/63 | S | G | T | . | V | I | R | R | V | . | . | V | M | . | A | G | . | V | . | . | . | . | . | N | . | * |

**Additional file 1f. Amino acid substitutions in the predicted M1 & M2 sequence compared to A/equine/New Market/3/05**

M1 Protein

M2 Protein

| *STRAIN* | *15* | *80* | *95* | *208* | *253* |
| --- | --- | --- | --- | --- | --- |
| New/5/03 | V | V | R | K |  |
| Rich/1/07 | I | I | K | . | * |
| Xinj/1/07 | I | I | K | . | * |
| Katr/6/08 | I | I | K | . | * |
| Myso/12/08 | I | I | K | . | * |
| Gope/1/09 | I | I | K | . | * |
| Xuzh/1/13 | I | I | K | . | * |
| Mong/11 | I | I | K | . | * |
| Heil/1/10 | I | I | K | . | * |
| Heil/10/08 | I | I | K | . | * |
| Gans/7/08 | I | I | K | . | * |
| Hube/6/08 | I | I | K | . | * |
| Inner-Mong/8/08 | I | I | K | . | * |
| Linc/1/07 | . | . | . | R | * |
| Kyon/1/11 | I | . | . | R | * |
| Czec/09 | . | . | . | R | * |
| Ohio/1/03 | . | . | . | R | * |
| Wisc/1/03 | . | . | . | R | * |
| kent/5/02 | . | . | . | . | * |
| Cali/8560/02 | . | . | . | . | * |
| New/1/93 | . | . | . | . | * |
| New/2/93 | . | . | . | . | * |
| Hong/1/92 | . | . | . | . | * |
| Suss/1/89 | . | . | . | . | * |
| Kent/1/81 | . | . | . | . | * |
| Font/1/79 | . | . | . | . | * |
| Miam/1/63 | . | . | . | Q | * |

| *STRAIN* | *17* | *21* | *48* | *85* | *89* | *98* |
| --- | --- | --- | --- | --- | --- | --- |
| New/5/03 | K | D | F | D | G | * |
| Rich/1/07 | . | . | . | S | S | * |
| Xinj/1/07 | . | G | S | S | S | * |
| Katr/6/08 |  | G | S | S | S | * |
| Myso/12/08 | N | G | S | S | S | * |
| Gope/1/09 | . | G | S | S | S | * |
| Xuzh/1/13 | . | G | S | N | S | * |
| Mong/1/11 | . | G | S | S | S | * |
| Heil/1/10 | N | G | S | S | S | * |
| Heil/10/08 | . | G | S | S | S | * |
| Gans/7/08 | . | G | S | S | S | * |
| Hube/6/08 | N | G | S | S | S | * |
| Inner-Mon/8/08 | N | G | S | S | S | * |
| Linc/1/07 | . | . | . | . | . | * |
| Kyon/1/11 | . | . | . | . | . | * |
| Czec/09 | . | . | . | . | . | * |
| Ohio/1/03 | . | . | . | . | . | * |
| Wisc/1/03 | . | . | . | . | . | * |
| Kent/5/02 | . | . | . | . | . | * |
| Cali/8560/02 | . | . | . | . | . | * |
| New/1/93 | . | . | . | . | . | * |
| New/2/93 | . | G | . | . | . | * |
| Hong/1/92 | . | . | . | . | . | * |
| Suss/1/89 | . | . | . | . | . | * |
| Kent/1/81 | . | . | . | . | . | * |
| Font/1/79 | . | . | . | . | . | * |
| Miam/1/63 | . | . | . | . | . | * |
