# Supplementary figures and images for: Genetic and codon usage bias analyses of polymerase genes of equine influenza virus and its relation to evolution
Source: BMC Genomics. 2017 Aug 23;18:652. doi: 10.1186/s12864-017-4063-1 (PMC5568313; doi:10.1186/s12864-017-4063-1)

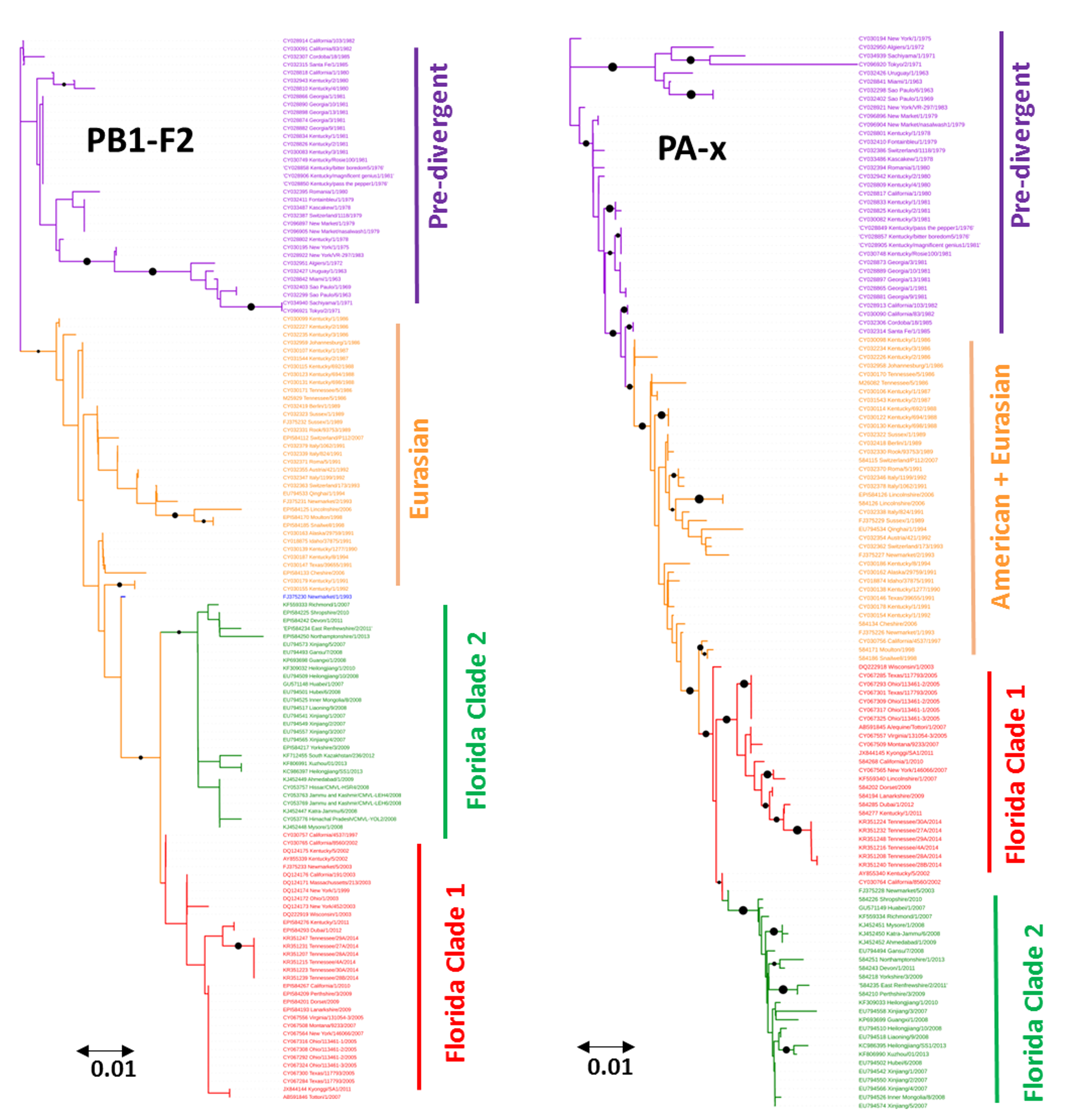

Supplement: Supplementary file 2 — PA-X and PB1-F2 phylograms. (TIFF 2690 kb) [file 12864_2017_4063_MOESM2_ESM.tif]

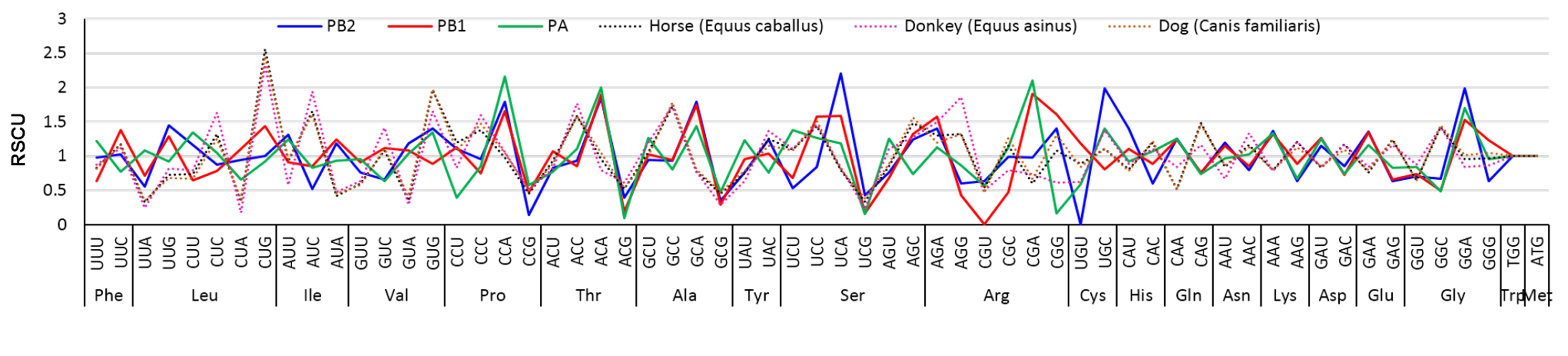

Supplement: Supplementary file 4 — Comparison of Relative Synonymous Codon Usage (RSCU) patterns of EIVs polymerase genes with their host species. (TIFF 1828 kb) [file 12864_2017_4063_MOESM4_ESM.tif]

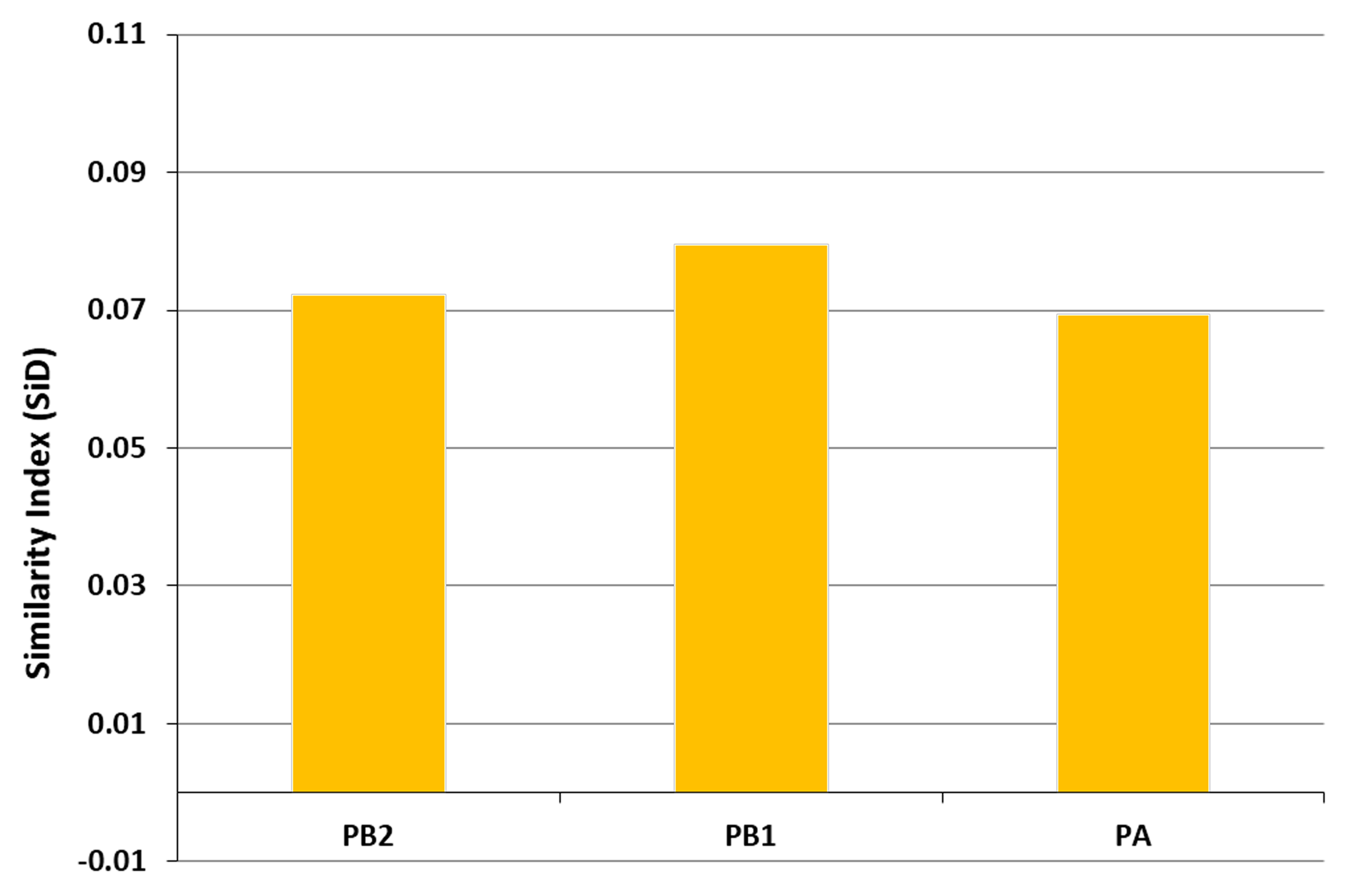

Supplement: Supplementary file 5 — The similarity index analysis of of EIVs polymerase genes in relation to their host species, Equus caballus. (TIFF 358 kb) [file 12864_2017_4063_MOESM5_ESM.tif]
