# Supplementary material for: Genetic and codon usage bias analyses of polymerase genes of equine influenza virus and its relation to evolution
Source: BMC Genomics. 2017 Aug 23;18:652. doi: 10.1186/s12864-017-4063-1 (PMC5568313; doi:10.1186/s12864-017-4063-1)
Supplement: Supplementary file 3 — Correlation coefficients among the position of genes along the first two major axes with various indexes of PA polymerase genes’ codon usage. (DOCX 33 kb) [file 12864_2017_4063_MOESM3_ESM.docx]

**Additional file 3**  Correlation coefficients among the position of genes along the first two major axes with various indexes of PA polymerase genes’ codon usage.

| **Variables** | **A(%)** | **C(%)** | **G(%)** | **U(%)** | **U3 (%)** | **C3(%)** | **A3(%)** | **G3(%)** | **%GC** | **%GC1** | **%GC2** | **%GC3** | **GC12** | **GC3s** | **ENc** | **CAI** | **Axis1** |
| --- | --- | --- | --- | --- | --- | --- | --- | --- | --- | --- | --- | --- | --- | --- | --- | --- | --- |
| **A(%)** |  |  |  |  |  |  |  |  |  |  |  |  |  |  |  |  |  |
| **C(%)** | -0.913** |  |  |  |  |  |  |  |  |  |  |  |  |  |  |  |  |
| **G(%)** | -0.889** | 0.747** |  |  |  |  |  |  |  |  |  |  |  |  |  |  |  |
| **U(%)** | 0.604** | -0.755** | -0.681** |  |  |  |  |  |  |  |  |  |  |  |  |  |  |
| **U3 (%)** | 0.560** | -0.651** | -0.688** | 0.923** |  |  |  |  |  |  |  |  |  |  |  |  |  |
| **C3(%)** | -0.880** | 0.948** | 0.755** | -0.774** | -0.737** |  |  |  |  |  |  |  |  |  |  |  |  |
| **A3(%)** | 0.961** | -0.878** | -0.850** | 0.573** | 0.552** | -0.850** |  |  |  |  |  |  |  |  |  |  |  |
| **G3(%)** | -0.782** | 0.672** | 0.899** | -0.635** | -0.706** | 0.674** | -0.843** |  |  |  |  |  |  |  |  |  |  |
| **%GC** | -0.968** | 0.947** | 0.896** | -0.728** | -0.667** | 0.926** | -0.920** | 0.784** |  |  |  |  |  |  |  |  |  |
| **%GC1** | -0.498** | 0.469** | 0.410** | -0.263* | -0.099* | 0.400** | -0.343* | 0.100 | 0.508** |  |  |  |  |  |  |  |  |
| **%GC2** | -0.642** | 0.676** | 0.553** | -0.543** | -0.426** | 0.570** | -0.592** | 0.498** | 0.635** | 0.066 |  |  |  |  |  |  |  |
| **%GC3** | -0.881** | 0.870** | 0.869** | -0.751** | -0.784** | 0.908** | -0.908** | 0.886** | 0.922** | 0.280* | 0.518** |  |  |  |  |  |  |
| **GC12** | -0.830** | 0.828** | 0.709** | -0.595** | -0.427** | 0.723** | -0.699** | 0.472** | 0.832** | 0.737** | 0.678** | 0.613** |  |  |  |  |  |
| **GC3s** | -0.875** | 0.879** | 0.851** | -0.753** | -0.774** | 0.905** | -0.909** | 0.880** | 0.917** | 0.257* | 0.546** | 0.995** | 0.611** |  |  |  |  |
| **ENc** | -0.611** | 0.536** | 0.541** | -0.332* | -0.389** | 0.542** | -0.684** | 0.645** | 0.573** | 0.003 | 0.312* | 0.631** | 0.286* | 0.630** |  |  |  |
| **CAI** | -0.779** | 0.767** | 0.798** | -0.718** | -0.693** | 0.762** | -0.771** | 0.780** | 0.809** | 0.215* | 0.610** | 0.820** | 0.606** | 0.826** | 0.361** |  |  |
| **Axis1** | 0.911** | -0.865** | -0.774** | 0.583** | 0.564** | -0.892** | 0.910** | -0.725** | -0.880** | -0.355** | -0.575** | -0.848** | -0.703** | -0.840** | -0.659** | -0.657** |  |
| **Axis2** | 0.409** | -0.353** | -0.160 | -0.133 | -0.210* | -0.327* | 0.399** | -0.067 | -0.329* | -0.277* | -0.278* | -0.217* | -0.344* | -0.221* | -0.022 | -0.132 | 0.469** |

**p < 0.0001, *p < 0.05

**Supplementary Table 2b. Correlation coefficients among the position of genes along the first two major axes with various indexes of PB1 polymerase genes’ codon usage.**

| **Variables** | **A(%)** | **C(%)** | **G(%)** | **U(%)** | **U3 (%)** | **C3(%)** | **A3(%)** | **G3(%)** | **%GC** | **%GC1** | **%GC2** | **%GC3** | **GC12** | **GC3s** | **ENc** | **CAI** | **Axis1** |
| --- | --- | --- | --- | --- | --- | --- | --- | --- | --- | --- | --- | --- | --- | --- | --- | --- | --- |
| **A(%)** |  |  |  |  |  |  |  |  |  |  |  |  |  |  |  |  |  |
| **C(%)** | -0.680** |  |  |  |  |  |  |  |  |  |  |  |  |  |  |  |  |
| **G(%)** | -0.985** | 0.657** |  |  |  |  |  |  |  |  |  |  |  |  |  |  |  |
| **U(%)** | 0.648** | -0.984** | -0.639** |  |  |  |  |  |  |  |  |  |  |  |  |  |  |
| **U3 (%)** | 0.707** | -0.969** | -0.698** | 0.973** |  |  |  |  |  |  |  |  |  |  |  |  |  |
| **C3(%)** | -0.732** | 0.976** | 0.709** | -0.950** | -0.980** |  |  |  |  |  |  |  |  |  |  |  |  |
| **A3(%)** | 0.981** | -0.695** | -0.961** | 0.660** | 0.714** | -0.746** |  |  |  |  |  |  |  |  |  |  |  |
| **G3(%)** | -0.978** | 0.681** | 0.974** | -0.657** | -0.709** | 0.725** | -0.992** |  |  |  |  |  |  |  |  |  |  |
| **%GC** | -0.941** | 0.807** | 0.925** | -0.788** | -0.836** | 0.852** | -0.937** | 0.929** |  |  |  |  |  |  |  |  |  |
| **%GC1** | -0.651** | 0.645** | 0.663** | -0.649** | -0.632** | 0.610** | -0.608** | 0.634** | 0.692** |  |  |  |  |  |  |  |  |
| **%GC2** | 0.783** | -0.551** | -0.757** | 0.503** | 0.564** | -0.606** | 0.852** | -0.846** | -0.747** | -0.520** |  |  |  |  |  |  |  |
| **%GC3** | -0.959** | 0.752** | 0.941** | -0.730** | -0.787** | 0.809** | -0.977** | 0.969** | 0.973** | 0.610** | -0.826** |  |  |  |  |  |  |
| **GC12** | 0.255* | 0.003 | -0.216* | -0.057 | -0.001 | -0.066 | 0.370** | -0.347** | -0.169 | 0.279* | 0.605** | -0.332* |  |  |  |  |  |
| **GC3s** | -0.958** | 0.727** | 0.937** | -0.688** | -0.735** | 0.771** | -0.971** | 0.961** | 0.939** | 0.631** | -0.830** | 0.963** | -0.315* |  |  |  |  |
| **ENc** | -0.706** | 0.281* | 0.718** | -0.278* | -0.324* | 0.324* | -0.665** | 0.677** | 0.590** | 0.386** | -0.415** | 0.614** | -0.104 | 0.613** |  |  |  |
| **CAI** | -0.788** | 0.839** | 0.782** | -0.803** | -0.862** | 0.891** | -0.794** | 0.782** | 0.844** | 0.593** | -0.706** | 0.831** | -0.176* | 0.820** | 0.301* |  |  |
| **Axis1** | -0.944** | 0.680** | 0.930** | -0.648** | -0.694** | 0.715** | -0.956** | 0.959** | 0.889** | 0.625** | -0.838** | 0.925** | -0.316* | 0.954** | 0.668** | 0.765** |  |
| **Axis2** | 0.331* | 0.192* | -0.345** | -0.169 | -0.173* | 0.193* | 0.326* | -0.357** | -0.160 | -0.060 | 0.233* | -0.234* | 0.229* | -0.249* | -0.678** | 0.232* | -0.353** |

**p < 0.0001, *p < 0.05

**Supplementary Table 2c. Correlation coefficients among the position of genes along the first two major axes with various indexes of PB2 polymerase genes’ codon usage.**

| **Variables** | **A(%)** | **C(%)** | **G(%)** | **U(%)** | **U3 (%)** | **C3(%)** | **A3(%)** | **G3(%)** | **%GC** | **%GC1** | **%GC2** | **%GC3** | **GC12** | **GC3s** | **ENc** | **CAI** | **Axis1** |
| --- | --- | --- | --- | --- | --- | --- | --- | --- | --- | --- | --- | --- | --- | --- | --- | --- | --- |
| **A(%)** |  |  |  |  |  |  |  |  |  |  |  |  |  |  |  |  |  |
| **C(%)** | -0.850** |  |  |  |  |  |  |  |  |  |  |  |  |  |  |  |  |
| **G(%)** | -0.987** | 0.837** |  |  |  |  |  |  |  |  |  |  |  |  |  |  |  |
| **U(%)** | 0.668** | -0.872** | -0.710** |  |  |  |  |  |  |  |  |  |  |  |  |  |  |
| **U3 (%)** | 0.654** | -0.851** | -0.693** | 0.949** |  |  |  |  |  |  |  |  |  |  |  |  |  |
| **C3(%)** | -0.851** | 0.954** | 0.856** | -0.856** | -0.892** |  |  |  |  |  |  |  |  |  |  |  |  |
| **A3(%)** | 0.959** | -0.854** | -0.955** | 0.684** | 0.683** | -0.881** |  |  |  |  |  |  |  |  |  |  |  |
| **G3(%)** | -0.940** | 0.873** | 0.954** | -0.753** | -0.752** | 0.893** | -0.979** |  |  |  |  |  |  |  |  |  |  |
| **%GC** | -0.978** | 0.909** | 0.983** | -0.776** | -0.762** | 0.913** | -0.953** | 0.957** |  |  |  |  |  |  |  |  |  |
| **%GC1** | -0.257* | 0.129 | 0.281* | -0.231* | -0.188* | 0.095 | -0.147 | 0.148 | 0.255* |  |  |  |  |  |  |  |  |
| **%GC2** | -0.419** | 0.285* | 0.399** | -0.198* | -0.121 | 0.233* | -0.299* | 0.268* | 0.377** | 0.083 |  |  |  |  |  |  |  |
| **%GC3** | -0.937** | 0.931** | 0.939** | -0.810** | -0.810** | 0.943** | -0.954** | 0.971** | 0.971** | 0.141 | 0.257* |  |  |  |  |  |  |
| **GC12** | -0.492** | 0.299* | 0.490** | -0.286* | -0.221* | 0.245* | -0.343* | 0.310* | 0.461** | 0.798** | 0.588** | 0.297* |  |  |  |  |  |
| **GC3s** | -0.880** | 0.917** | 0.881** | -0.814** | -0.789** | 0.902** | -0.903** | 0.933** | 0.915** | 0.079 | 0.249* | 0.954** | 0.239* |  |  |  |  |
| **ENc** | -0.861** | 0.851** | 0.853** | -0.688** | -0.679** | 0.849** | -0.877** | 0.887** | 0.880** | 0.035 | 0.175 | 0.906** | 0.186* | 0.895** |  |  |  |
| **CAI** | -0.929** | 0.861** | 0.933** | -0.748** | -0.729** | 0.865** | -0.924** | 0.943** | 0.937** | 0.140 | 0.337* | 0.939** | 0.338* | 0.912** | 0.823** |  |  |
| **Axis1** | 0.844** | -0.806** | -0.854** | 0.745** | 0.727** | -0.821** | 0.852** | -0.871** | -0.859** | -0.216* | -0.221* | -0.876** | -0.329* | -0.880** | -0.840** | -0.885** |  |
| **Axis2** | -0.904** | 0.891** | 0.912** | -0.788** | -0.779** | 0.900** | -0.921** | 0.946** | 0.932** | 0.131 | 0.270* | 0.952** | 0.301* | 0.952** | 0.851** | 0.941** | -0.882** |

**p < 0.0001, *p < 0.05
