# Supplementary material for: Genetic and codon usage bias analyses of polymerase genes of equine influenza virus and its relation to evolution
Source: BMC Genomics. 2017 Aug 23;18:652. doi: 10.1186/s12864-017-4063-1 (PMC5568313; doi:10.1186/s12864-017-4063-1)
Supplement: Supplementary file 6 — Correlation analysis among length (amino acid), GRAVY, AROMO, ENC, GC3s, ENc and the first two principle axes of COA of PB1 gene; Table S6b. Correlation analysis among length (amino acid), GRAVY, AROMO, ENC, GC3s, ENc and the first two principle axes of COA of PB2 gene; Table S6c. Correlation analysis among length (amino acid), GRAVY, AROMO, ENC, GC3s, ENc and the first two principle axes of COA of PA gene. (DOCX 18 kb) [file 12864_2017_4063_MOESM6_ESM.docx]

**Additional file 6a**  Correlation analysis among length (aa), GRAVY, AROMO, ENC, GC3s, ENc and the first two principle axes of COA of PA gene.

| **Variables** | **L_aa** | **GC** | **GC3s** | **ENc** | **Gravy** | **Aromo** | **Axis1** |
| --- | --- | --- | --- | --- | --- | --- | --- |
| **L_aa** |  |  |  |  |  |  |  |
| **GC** | 0.126 |  |  |  |  |  |  |
| **GC3s** | 0.090 | 0.917** |  |  |  |  |  |
| **ENc** | -0.042 | 0.573** | 0.630** |  |  |  |  |
| **Gravy** | -0.126 | 0.613** | 0.641** | 0.492** |  |  |  |
| **Aromo** | -0.302* | -0.761** | -0.753** | -0.407** | -0.442** |  |  |
| **Axis1** | -0.056 | -0.880** | -0.840** | -0.659** | -0.697** | 0.689** |  |
| **Axis2** | -0.039 | -0.329* | -0.221* | -0.022 | -0.317* | 0.040 | 0.469** |

**p < 0.0001, *p < 0.05

**Additional file 6b** Correlation analysis among length (aa), GRAVY, AROMO, ENC, GC3s, ENc and the first two principle axes of COA of PB1 gene.

| **Variables** | **GC** | **GC3s** | **ENc** | **Gravy** | **Aromo** | **Axis1** |
| --- | --- | --- | --- | --- | --- | --- |
| **GC** |  |  |  |  |  |  |
| **GC3s** | 0.939** |  |  |  |  |  |
| **ENc** | 0.590** | 0.613** |  |  |  |  |
| **Gravy** | -0.505** | -0.466** | -0.287* |  |  |  |
| **Aromo** | 0.371** | 0.326* | -0.145 | -0.305* |  |  |
| **Axis1** | 0.889** | 0.954** | 0.668** | -0.429** | 0.237* |  |
| **Axis2** | -0.160 | -0.249* | -0.678** | -0.085 | 0.531** | -0.353** |

**p < 0.0001, *p < 0.05

**Additional file 6c**  Correlation analysis among length (aa), GRAVY, AROMO, ENC, GC3s, ENc and the first two principle axes of COA of PB2 gene.

| **Variables** | **L_aa** | **GC** | **GC3s** | **ENc** | **Gravy** | **Aromo** | **Axis1** |
| --- | --- | --- | --- | --- | --- | --- | --- |
| **L_aa** |  |  |  |  |  |  |  |
| **GC** | 0.045 |  |  |  |  |  |  |
| **GC3s** | 0.020 | 0.915** |  |  |  |  |  |
| **ENc** | 0.010 | 0.880** | 0.895** |  |  |  |  |
| **Gravy** | 0.026 | -0.387** | -0.393** | -0.388** |  |  |  |
| **Aromo** | -0.224* | -0.120 | -0.057 | 0.059 | -0.031 |  |  |
| **Axis1** | -0.019 | -0.859** | -0.880** | -0.840** | 0.320* | 0.096 |  |
| **Axis2** | 0.016 | 0.932** | 0.952** | 0.851** | -0.400** | -0.055 | -0.882** |

**p < 0.0001, *p < 0.05
