# Supplementary material for: Genetic and codon usage bias analyses of polymerase genes of equine influenza virus and its relation to evolution
Source: BMC Genomics. 2017 Aug 23;18:652. doi: 10.1186/s12864-017-4063-1 (PMC5568313; doi:10.1186/s12864-017-4063-1)
Supplement: Supplementary file 7 — Primers used for amplification of polymerase genes of EIV. (DOCX 12 kb) [file 12864_2017_4063_MOESM7_ESM.docx]

**Additional file 7** Primers used for amplification of polymerase genes of EIV

| **Gene** | **Primer sequence** | **Amplicon (bp)** |
| --- | --- | --- |
| **PB2 (N-terminal region)** | PB2a-F:ATGGAGAGAATAAAAGAACT GAG  PB2a-R: CGC TGATTTGCTCTATTAAC | 1280 |
| **PB2 (C-terminal region)** | PB2b-F:GTAGCCATGGTGTTTTCGCAAGAAG  PB2b-R:CTAATTGATGGCCATCCGAATCCTTTT GG | 1083 |
| **PB1 (N-terminal region)** | PB1a-F:ATGGATGTCAATCCGACTCTAC  PB1a-R:TCCCACCAGTATGTGGTCTTTGTG | 1316 |
| **PB1 (C-terminal region)** | PB1b-F:CACTGAGTCCTGGCATGATGATGG  PB1b-R:CTATTTTTGCCGTCTGAGCTCTTCAATGG | 1070 |
| **PA (N-terminal region)** | PAa-F:ATGGAAGACTTTGTGCGAC  PAa-R: CCAATTTCATCGAGCTCTATCCAGC | 1286 |
| **PA (C-terminal region)** | PAb-F:AGGTCTCTTGCAAGTTGG  PAb-R: CTACTTCAGTGCATGTGTAAGGAAGG | 951 |
