# Supplementary material for: Genetic and codon usage bias analyses of polymerase genes of equine influenza virus and its relation to evolution
Source: BMC Genomics. 2017 Aug 23;18:652. doi: 10.1186/s12864-017-4063-1 (PMC5568313; doi:10.1186/s12864-017-4063-1)
Supplement: Supplementary file 8 — Details of polymerase gene sequences of Equine Influenza virus (EIV) strains of equid origin used in the study. (DOC 69 kb) [file 12864_2017_4063_MOESM8_ESM.doc]

**Additional file 8** Details of polymerase gene sequences of Equine Influenza virus (EIV) strains of equid origin used in the study.

| **EIV Isolates** | **Country** | **Year** | **Accession nos** |
| --- | --- | --- | --- |
| A/equine/Richmond/1/07 | UK | 2007 | KF559332 (PB2), KF559333(PB1), KF559334(PA) |
| A/equine/Xinjiang/1/07 | China | 2007 | EU794540 (PB2), EU794541(PB1), EU794542(PA) |
| A/equine/Katra-Jammu/6/08 | India | 2008 | KJ452444 (PB2), KJ452447(PB1), KJ452447(PA) |
| A/equine/Mysore/1/08 | India | 2008 | KJ452445 (PB2), KJ452448(PB1), KJ452451(PA) |
| A/equine/Gansu/7/08 | China | 2008 | EU794492(PB2), EU794493(PB1), EU794494(PA) |
| A/equine/Hubei/6/08 | China | 2008 | EU794500(PB2), EU794501(PB1), EU794502(PA) |
| A/equine/Inner Mongolia/8/08 | China | 2008 | EU794524 (PB2), EU794525(PB1), EU794526(PA) |
| A/equine/Ahmedabad/1/09 | India | 2009 | KJ452446 (PB2), KJ452449(PB1), KJ452452(PA) |
| A/equine/Yorkshire/3/09 | UK | 2009 | EPI584216 (PB2), EPI584217 (PB1), EPI584218(PA) |
| A/equine/Perthshire/3/09 | UK | 2009 | EPI584208 (PB2), EPI584209 (PB1), EPI584210 (PA) |
| A/equine/Heilongjiang/1/10 | China | 2010 | KF309031 (PB2), JX629296(PB1), KF309033(PA) |
| A/equine/Shropshire/10 | UK | 2010 | EPI584224 (PB2), EPI584225 (PB1), EPI584226 (PA) |
| A/equine/Devon/1/11 | UK | 2011 | EPI584241 (PB2), EPI584242 (PB1), EPI584243 (PA) |
| A/equine/East_Renfrewshire/2/11 | Scotland | 2011 | EPI584233 (PB2), EPI584234 (PB1), EPI584235 (PA) |
| A/equine/Dubai/1/12 | UAE | 2012 | EPI584292 (PB2), EPI584293 (PB1), EPI584294 (PA) |
| A/equine/Xuzhou/01/13 | China | 2013 | KF806992 (PB2), KF806991(PB1), KF806990(PA) |
| A/equine/Northamptonshire/1/13 | UK | 2013 | EPI584249 (PB2), EPI584250 (PB1), EPI584251 (PA) |
| A/equine/Newmarket/5/03 | UK | 2003 | FJ375221 (PB2), FJ375233(PB1), FJ375228(PA) |
| A/equine/Athens/4/07 | Greece | 2007 | HQ696125(PB2), HQ696123(PB1), HQ696121(PA) |
| A/equine/Athens/2/03 | Greece | 2003 | HQ696124(PB2), HQ696122(PB1), HQ696120(PA) |
| A/equine/Lanarkshire/09 | UK | 2009 | EPI584192 (PB2), EPI584193 (PB1), EPI584194 (PA) |
| A/equine/Dorset/09 | Dorset | 2009 | EPI584200 (PB2), EPI584201 (PB1), EPI584202 (PA) |
| A/equine/California/1/10 | USA | 2010 | EPI584266 (PB2), EPI584267 (PB1), EPI584268 (PA) |
| A/equine/Kyonggi/SA1/11 | South Korea | 2011 | JX844143(PB2), JX844144(PB1), JX844144((PA) |
| A/equine/Kentucky/1/11 | USA | 2011 | EPI584275 (PB2), EPI584276 (PB1), EPI584277 (PA) |
| A/equine/Lincolnshire/1/07 | UK | 2007 | KF559338(PB2), KF559339(PB1), KF559340(PA) |
| A/equine/Almaty/26/07 | Kazakhstan | 2007 | JX306642(PB2), JX306641(PB1), JX306640(PA) |
| A/equine/Montana/9233/07 | USA | 2007 | CY067507(PB2), CY067508(PB1), CY067509(PA) |
| A/equine/Cheshire/06 | UK | 2006 | EPI584132 (PB2), EPI584133 (PB1) EPI584134 (PA) |
| A/equine/Lincolnshire/06 | UK | 2006 | EPI584124 (PB2), EPI584125 (PB1) EPI584126 (PA) |
| A/equine/Wisconsin/1/03 | USA | 2003 | DQ222920(PB2), DQ222919(PB1), DQ222918(PA) |
| A/equine/Ohio/113461-3/05 | USA | 2005 | CY067291(PB2), CY067324(PB1), CY067323(PA) |
| A/equine/Kentucky/5/02 | USA | 2002 | AY855338 (PB2), AY855339(PB1), AY855340(PA) |
| A/equine/Snailwell/98 | UK | 1998 | EPI584184 (PB2), EPI584185 (PB1) EPI584186 (PA) |
| A/equine/Kentucky/8/94 | USA | 1994 | CY030188(PB2), CY030187(PB1), CY030186(PA) |
| A/equine/Newmarket/1/93 | UK | 1993 | FJ375218(PB2), FJ375230(PB1), FJ375226(PA) |
| A/equine/Newmarket/2/93 | UK | 1993 | FJ375219(PB2), FJ375231(PB1), FJ375227(PA) |
| A/equine/Switzerland/173/93 | Switzerland | 1993 | CY032364(PB2), CY032363(PB1), CY032362(PA) |
| A/equine/Kentucky/1/92 | USA | 1992 | CY030156 (PB2), CY030155(PB1), CY030154(PA) |
| A/equine/Italy/1199/92 | Italy | 1992 | CY032348(PB2), CY032347(PB1), CY032346(PA) |
| A/equine/Kentucky/1/91 | USA | 1991 | CY030180(PB2), CY030179(PB1), CY030178(PA) |
| A/equine/Alaska/29759/91 | USA | 1991 | CY030164 (PB2), CY030163(PB1), CY030162(PA) |
| A/equine/Kentucky/1277/90 | USA | 1990 | CY030140(PB2), CY030139(PB1), CY030138(PA) |
| A/equine/Sussex/1/89 | UK | 1989 | FJ375220(PB2), FJ375232(PB1), FJ375229(PA) |
| A/equine/Kentucky/692/88 | USA | 1988 | CY030116(PB2), CY030115(PB1), CY030114(PA) |
| A/equine/Kentucky/1/87 | USA | 1987 | CY030108(PB2), CY030107(PB1), CY030106(PA) |
| A/equine/Johannesburg/1/86 | SA | 1986 | CY032960(PB2), CY032959(PB1), CY032958(PA) |
| A/equine/Kentucky/1/86 | USA | 1986 | CY030100(PB2), CY030099(PB1), CY030098(PA) |
| A/equine/Santa_Fe/1/85 | Mexico | 1985 | CY032316(PB2), CY032315(PB1), CY032314(PA) |
| A/equine/Kentucky/1/81 | USA | 1981 | CY028835(PB2), CY028834(PB1), CY028833(PA) |
| A/equine/Kentucky/2/80 | USA | 1980 | CY032944(PB2), CY032943(PB1), CY032942(PA) |
| A/equine/Fontainbleu/1/79 | France | 1979 | CY032412(PB2), CY032411(PB1), CY032410(PA) |
| A/equine/Sao_Paulo/1/69 | Brazil | 1969 | CY032404(PB2), CY032403(PB1), CY032402(PA) |
| A/equine/Uruguay/1/63 | South America | 1963 | CY032428(PB2), CY032427(PB1), CY032426(PA) |
| A/equine/Miami/1/63 | USA | 1963 | CY028843(PB2), CY028842(PB1), CY028841(PA) |

Note: UK: United Kingdom, USA: United States of America, SA: South Africa, UAE: United Arab Emirates
